# Variation in plant Toll/Interleukin-1 receptor domain protein dependence on *ENHANCED DISEASE SUSCEPTIBILITY 1*

**DOI:** 10.1093/plphys/kiac480

**Published:** 2022-10-13

**Authors:** Oliver Johanndrees, Erin L Baggs, Charles Uhlmann, Federica Locci, Henriette L Läßle, Katharina Melkonian, Kiara Käufer, Joram A Dongus, Hirofumi Nakagami, Ksenia V Krasileva, Jane E Parker, Dmitry Lapin

**Affiliations:** Department of Plant-Microbe Interactions, Max Planck Institute for Plant Breeding Research, Cologne, Germany; Department of Plant and Microbial Biology, University of California Berkeley, Berkeley, California, USA; Earlham Institute, Norwich Research Park, Norwich, UK; Department of Plant-Microbe Interactions, Max Planck Institute for Plant Breeding Research, Cologne, Germany; Department of Plant-Microbe Interactions, Max Planck Institute for Plant Breeding Research, Cologne, Germany; Department of Plant-Microbe Interactions, Max Planck Institute for Plant Breeding Research, Cologne, Germany; Department of Plant-Microbe Interactions, Max Planck Institute for Plant Breeding Research, Cologne, Germany; Department of Plant-Microbe Interactions, Max Planck Institute for Plant Breeding Research, Cologne, Germany; Department of Plant-Microbe Interactions, Max Planck Institute for Plant Breeding Research, Cologne, Germany; Department of Plant-Microbe Interactions, Max Planck Institute for Plant Breeding Research, Cologne, Germany; Department of Plant and Microbial Biology, University of California Berkeley, Berkeley, California, USA; Earlham Institute, Norwich Research Park, Norwich, UK; Department of Plant-Microbe Interactions, Max Planck Institute for Plant Breeding Research, Cologne, Germany; Cluster of Excellence on Plant Sciences (CEPLAS), Düsseldorf, Germany; Department of Plant-Microbe Interactions, Max Planck Institute for Plant Breeding Research, Cologne, Germany; Department of Biology, Translational Plant Biology, Utrecht University, Utrecht, The Netherlands

## Abstract

Toll/Interleukin-1 receptor (TIR) domains are integral to immune systems across all kingdoms. In plants, TIRs are present in nucleotide-binding leucine-rich repeat (NLR) immune receptors, NLR-like, and TIR-only proteins. Although TIR-NLR and TIR signaling in plants require the ENHANCED DISEASE SUSCEPTIBILITY 1 (EDS1) protein family, TIRs persist in species that have no EDS1 members. To assess whether particular TIR groups evolved with EDS1, we searched for TIR-EDS1 co-occurrence patterns. Using a large-scale phylogenetic analysis of TIR domains from 39 algal and land plant species, we identified 4 TIR families that are shared by several plant orders. One group occurred in TIR-NLRs of eudicots and another in TIR-NLRs across eudicots and magnoliids. Two further groups were more widespread. A conserved TIR-only group co-occurred with EDS1 and members of this group elicit *EDS1*-dependent cell death. In contrast, a maize (*Zea mays*) representative of TIR proteins with tetratricopeptide repeats was also present in species without EDS1 and induced *EDS1*-independent cell death. Our data provide a phylogeny-based plant TIR classification and identify TIRs that appear to have evolved with and are dependent on *EDS1*, while others have *EDS1*-independent activity.

## Teaser

Plant Toll/Interleukin-1 receptor domain proteins can use different mechanisms to induce cell death.

## Introduction

Toll/Interleukin-1 receptor (TIR) domains regulate immune signaling and cell death in bacteria, animals, and plants (Nimma et al., 2017; Essuman et al., 2022; Lapin et al., 2022). In bacteria, TIR domain proteins constitute antiphage defense systems or act as virulence factors (Coronas-Serna et al., 2020; Morehouse et al., 2020; Eastman et al., 2021; Ofir et al., 2021). In animals, TIRs function as signal transduction modules within specialized adapters (e.g. myeloid differentiation primary response 88 [MyD88]) and in receptor proteins such as Toll-like receptors (TLRs) and sterile alpha and TIR motif-containing protein 1 (SARM1), which sense pathogen-associated molecular patterns (PAMPs) and cell metabolic changes, respectively (O'Neill and Bowie, 2007; Figley et al., 2021; Shi et al., 2022). In plants, intracellular immune receptors with N-terminal TIR domains have a central domain called nucleotide-binding adaptor (NB) shared by APAF-1, certain *R*-gene products and CED-4 (NBARC), and C-terminal leucine-rich repeats (LRRs) (van der Biezen and Jones, 1998). This receptor class (referred to as TIR-NLR or TNL) detects pathogen virulence factor (effector) activities to induce defenses which often culminate in localized host cell death (Jones et al., 2016; Lapin et al., 2022). Several plant-truncated TIR-only and TIR-NBARC proteins also contribute to pathogen detection or defense amplification (Nandety et al., 2013; Nishimura et al., 2017; Tian et al., 2021; Lapin et al., 2022; Yu et al., 2022). No functional TIR adapters were found in plants to date.

Interactions between activated animal TLRs and TIR adapter proteins transduce pathogen recognition into defense via protein kinase activation and transcriptional reprogramming (Fields et al., 2019; Clabbers et al., 2021). Bacterial pathogens of mammals utilize TIR effector hetero-dimerization with host TIRs to disrupt MyD88-mediated TLR signaling (Cirl et al., 2008; Yadav et al., 2010; Nanson et al., 2020). Another mechanism was discovered in human SARM1, in which TIRs hydrolyze nicotinamide adenine dinucleotide (NAD^+^) leading to neuronal cell death (Gerdts et al., 2015; Essuman et al., 2017; Horsefield et al., 2019; Sporny et al., 2019; Shi et al., 2022). NAD^+^ cleavage activity was found in TIRs of the bacterial antiphage Thoeris system, TIR-STING cyclic dinucleotide receptors (Morehouse et al., 2020; Ofir et al., 2021), bacterial TIR effectors (Coronas-Serna et al., 2020; Eastman et al., 2021), plant TNLs, and TIR-only proteins (Horsefield et al., 2019; Wan et al., 2019; Ma et al., 2020). TIR NADase activity and associated host cell death require a conserved catalytic glutamate residue in a pocket formed by self-associating TIRs (Essuman et al., 2017, 2018; Horsefield et al., 2019; Wan et al., 2019; Ma et al., 2020; Martin et al., 2020; Burdett et al., 2021; Lapin et al., 2022). Some plant TIR domains are bifunctional enzymes with the capacity for 2′,3′-cAMP/cGMP synthetase activity which potentiates cell death. The same catalytic glutamate residue was important for both TIR enzymatic activities (Yu et al., 2022). Thus, TIRs display enzymatic and functional versatility (Essuman et al., 2022; Lapin et al., 2022; Yu et al., 2022).

Previously, TIRs in prokaryotes and eukaryotes were divided into 37 groups through Bayesian partitioning with pattern selection (BPPS) (Toshchakov and Neuwald, 2020). The majority of plant TIRs were assigned to three plant-specific groups following domain architectures of the full-length proteins, although approximately 1,000 plant TIRs remain unclassified (Toshchakov and Neuwald, 2020). The largest plant-specific group was enriched for TIRs from TNLs, and the two remaining groups included TIR-only proteins and TIRs fused to NBARC-like domains (Toshchakov and Neuwald, 2020). The latter group corresponds to the so-called XTNX proteins, where X indicates conserved N-terminal and C-terminal sequences (Meyers et al., 2002; Nandety et al., 2013; Zhang et al., 2017a, 2017b). Because XTNXs contain tetratricopeptide-like repeats (TPRs) instead of LRRs (reviewed in Lapin et al., 2022), originally described in this study), we call XTNXs from herein TIR-NBARC-TPRs (TNPs), to reflect their domain architecture, fitting with the existing NLR nomenclature. The BPPS grouping of plant TIRs aligns with earlier studies employing phylogeny-based group assignment of TIRs (Meyers et al., 2002; Nandety et al., 2013).

In eudicot plants, all tested TIR-only and TNL proteins function via a plant-specific protein family comprising ENHANCED DISEASE SUSCEPTIBILITY 1 (EDS1), PHYTOALEXIN-DEFICIENT 4 (PAD4), and SENESCENCE-ASSOCIATED GENE 101 (SAG101) (Lapin et al., 2020; Dongus and Parker, 2021). The EDS1 family proteins contain an N-terminal lipase-like domain and C-terminal α-helical bundle EDS1–PAD4 domain (EP, PFAM: PF18117) which, together, characterize the EDS1 family (Wagner et al., 2013; Baggs et al., 2020; Lapin et al., 2020). EDS1 forms a dimer with either PAD4 or SAG101 to mediate pathogen resistance and cell death triggered by plant TIRs (Wagner et al., 2013; Bhandari et al., 2019; Gantner et al., 2019; Lapin et al., 2019; Sun et al., 2021; Dongus et al., 2022). The EDS1 family coevolved and cofunctions with two conserved coiled-coil domain NLR groups ACTIVATED DISEASE RESISTANCE 1 (ADR1) and N REQUIREMENT GENE 1 (NRG1) (Collier et al., 2011; Lapin et al., 2019; Baggs et al., 2020; Saile et al., 2020; Sun et al., 2021; Wu et al., 2022). It is now known that EDS1–PAD4 and EDS1–SAG101 heterodimers serve as receptors for specific nucleotide-based plant TIR NADase products, which induce the dimer associations, respectively, with ADR1- and NRG1-type NLRs to promote immunity and/or host cell death (Essuman et al., 2022; Huang et al., 2022; Jia et al., 2022). In contrast, expression of the human SARM1 TIR domain or *Pseudomonas syringae* HopAM1 TIR effector-triggered *EDS1*-independent cell death in *Nicotiana benthamiana* (*Nb*) (Horsefield et al., 2019; Wan et al., 2019; Eastman et al., 2021), suggesting a degree of specificity in translating TIR catalytic activity into immune responses via the EDS1 family (Lapin et al., 2022). Consistent with plant EDS1 family—TIR cofunctions, expanded TNL repertoires are found in seed plants with the EP domain sequences (Wagner et al., 2013; Lapin et al., 2019; Baggs et al., 2020; Liu et al., 2021). However, the existence of TNPs and other TIRs in plant genomes that lack *EDS1* (Meyers et al., 2002; Gao et al., 2018; Toshchakov and Neuwald, 2020; Lapin et al., 2022) raises the question of whether a subset of plant TIRs function in an *EDS1*-independent manner.

Our aim was to find signatures of EDS1-TIR co-occurrence which could be used to predict EDS1 dependency of distinct TIR groups in plants. By phylogeny-based clustering of predicted TIR sequences from 39 species representing diverse taxons of green plants, we identify 4 TIR groups that are shared by at least two plant lineages. Two of these groups match TIRs of the previously identified TNPs and conserved TIR-only proteins (Meyers et al., 2002; Nandety et al., 2013; Lapin et al., 2022). Two other TIR groups are nested within angiosperm TNLs. *Nb* mutants for *TNP*s, encoding the most conserved and widely distributed TIR proteins in plants, behave like wild-type (WT) plants in tested PAMP-triggered and TNL immunity outputs. We further establish that a TNP from maize (*Zea mays*) elicits *EDS1*-independent cell death in tobacco (*Nicotiana tabacum*) transient expression assays. Conversely, immunity-induced expression of the conserved *TIR-only* genes, *EDS1* dependency of cell death elicited by these proteins in *Nb*, and their co-occurrence with EDS1/PAD4/ADR1 suggest the importance of an EDS1/PAD4/ADR1—conserved TIR-only signaling node in the immune system of flowering plants. Hence, there appears to be selectivity at the level of EDS1 by plant TIRs for cell death activity.

## Results

### Land plants have four taxonomically shared TIR groups

To study the distribution of TIRs in plants, we utilized predicted protein sequences from 39 species comprising unicellular green algae, nonseed land plants, conifers, and 7 clades of flowering plants (*Amborella trichopoda* or *Amborella* here on, *Nymphaeales*, *Magnoliids*, *Ceratophyllales*, monocots, superrosids, and superasterids) (Supplemental Table S1). In total, 2,348 TIRs were predicted using hidden Markov models (HMMs; Supplemental Table S2). The number of predicted TIR-containing sequences per plant species ranged from a single protein in common liverwort (*Marchantia polymorpha*) (Bowman et al., 2017) and gemniferous spikemoss (*Selaginella moellendorffii*) to 435 and 477 in the Rosid flooded gum (*Eucalyptus grandis*) and conifer loblolly pine (*Pinus taeda*), respectively. Generally, the highest numbers of predicted TIR-containing proteins were found in eudicots (Supplemental Figure S1A; Sun et al., 2014; Liu et al., 2021). Analyses of the protein domain composition revealed 1,020 TNLs, 401 TN, and 572 TIR-only architectures (Supplemental Figure S1, B–D; Sun et al., 2014). As expected, TNLs were missing in monocots and seep monkey-flowers (*Erythranthe guttata*; Shao et al., 2016; Liu et al., 2021). Low TNL numbers were found in two *Caryophyllales* (prince’s feather [*Amaranthus hypochondriacus*] and sugar beet [*Beta vulgaris*]) (Shao et al., 2016; Lapin et al., 2019; Baggs et al., 2020; Liu et al., 2021). Whereas TNLs were found in 20 of 39 analyzed species, TIR-only proteins (sequences shorter than 400 amino acids and without other predicted PFAM domains) were present in 33 of the 39 species, including unicellular green algae and monocots (Supplemental Figure S1D; Sun et al., 2014; Liu et al., 2021). Thus, TIR-only is likely the most widely adopted TIR protein architecture across land plants and green algae.

**Figure 1:**
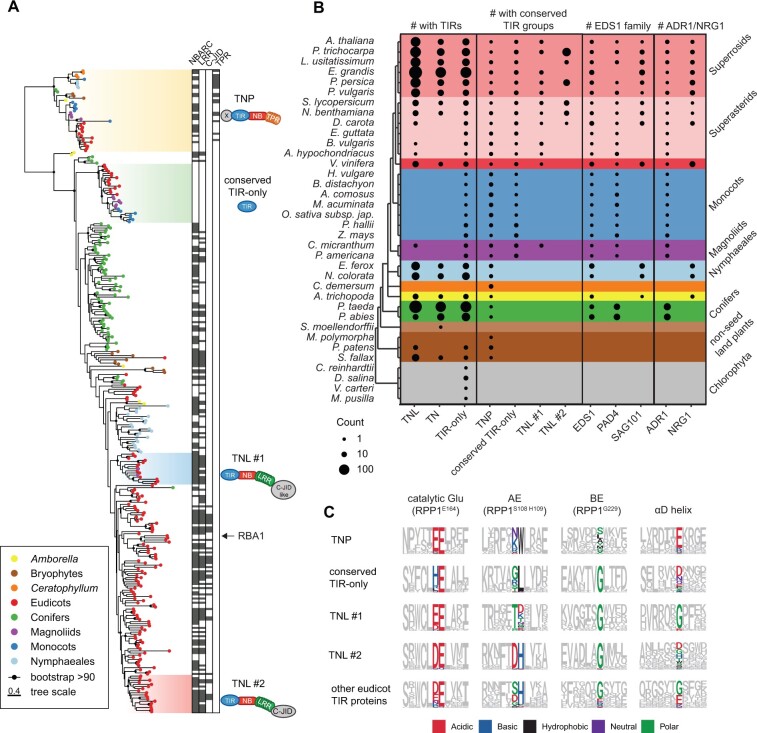
Land plants share four TIR groups. A, ML tree (evolutionary model WAG+F+R7) of 307 predicted TIR domain sequences representing major TIR families across plant species (full 2,317 sequence tree in Supplemental Figure S2B). Branches with BS support ≥90% are marked with black dots. Taxonomically shared TIR groups from more than one order are highlighted with colored boxes and their predominant domain architecture is depicted. Additional domains predicted in the TIR proteins are annotated as black boxes next to each TIR protein (used HMMs listed in Supplemental Table S2). Four TIR domain groups shared by at least two taxonomic groups (e.g. Rosids and Asterids in the case of TNL#2) were named after the predominant domain architecture of full-length proteins. The presence of TPRs in TNPs was deduced based on the TPR HMMs (Supplemental Table S2). The TIR-only RBA1/*At*TX1 does not belong to conserved TIR-only proteins. The scale bar corresponds to number of substitutions per site. B, Counts of predicted full-length TIR proteins, proteins with taxonomically shared TIRs, ADR1/NRG1, and EDS1 family predicted in the species analyzed in this study. TNPs are not included in the counts of TNL, TN, and TIR-only proteins. TIR-only proteins are defined as sequences shorter than 400 amino acids, without other predicted PFAM domains. Sizes of circles reflect the counts. *Eucalyptus grandis* has a fragment of PAD4-like sequence as determined by TBLASTN searches. C, Comparison of important TIR domain motifs across the four conserved plant TIR groups. Full sets of TIR domains were taken based on phylogeny (tree in Supplemental Figure S2B). Sequence motifs were generated for each TIR group to show conservation of the catalytic glutamate, AE, and BE interfaces, as well as residues in the αD helix. *Arabidopsis thaliana* RPP1^WsB^ TIR domain was taken as reference. Chemical attributes of the important amino acids are annotated in different colors. C-JID, C-terminal jelly roll/Ig-like domain; NBARC, nucleotide-binding domain shared by APAF-1, certain *R*-gene products, and CED-4; RBA1, RECOGNITION OF HOPBA1. Full species names are in Supplemental Table S1.

To categorize plant TIRs based on their sequence rather than just the protein domain architecture, we constructed a maximum likelihood (ML) phylogenetic tree for the 2,348 TIR sequences (Supplemental Figure S2A; Supplemental Files S1 and S2). This analysis revealed four TIR groups supported with ultrafast bootstrap values >90% and shared by several taxonomic groups higher than order, for instance by Rosids and Asterids (“taxonomically shared TIR groups”). Algal sequences did not form a monophyletic group and did not fall into the four shared TIR groups. Since algal TIR sequences tended to have long branches, we excluded them from further analysis and repeated the ML tree inference for the remaining 2,317 sequences (Supplemental Figure S2B and Supplemental Files S3–S5). The same four phylogenetically distinct TIR groups were shared by land plant lineages. A large excess of sequences over the number of alignment patterns can lead to false phylogenetic inferences. Therefore, we prepared a reduced ML tree for 307 representative TIRs (Figure 1A) selected from the major groups on the bigger ML tree (Supplemental Figure S2C and Supplemental Files S6 and S7). The same four TIR groups were recovered again, despite different alignments and underlying evolutionary models (Figure 1A; BS > 90%, SH-aLRT > 80). Since NBARC domain types match with NLR classes (Shao et al., 2016; Tamborski and Krasileva, 2020), we tested whether the TIR groups identified here are associated with different NBARC variants. For that, we constructed an ML phylogenetic tree for associated NBARCs from full-length TIR-containing sequences used in Figure 1A (Supplemental Figure S3 and Supplemental Files S8 and S9). NBARCs linked with the above TIR groups also formed well-supported branches (BS > 90%, SH-aLRT > 80), suggesting that these TIRs have coevolved with their NBARCs. We conclude that land plants have four phylogenetically distinct TIR groups shared by at least two taxonomic clades.

### Taxonomically shared TIRs coincide with different protein domain architectures

Next, we investigated whether full-length proteins with taxonomically shared TIRs have specific domain architectures and how these align with earlier studies. Two TIR groups match two TNL families. One is also known as a “conserved TNL lineage” or “NLR family 31” in studies deploying NBARC phylogeny and synteny searches (Zhang et al., 2016; Liu et al., 2021). We use the term TNL #1 hereafter for this TNL group. Although the post-LRR C-terminal extension in TNL #1 proteins does not show similarity to other PFAM domains, AlphaFold2-predicted structures of Arabidopsis (*Arabidopsis thaliana*) TNL #1 proteins (AF-F4HR53-F1 and AF-F4HR54-F1) have a β-sandwich similar to C-terminal jelly-roll/Ig-like domain (C-JID, PF20160) from TNLs RECOGNITION OF *PERONOSPORA PARASITICA* 1 (RPP1^WsB^) and Recognition of XopQ 1 (Roq1) (Dali scores > 7.0) (Dodds et al., 2001; Van Ghelder and Esmenjaud, 2016; Holm, 2020; Ma et al., 2020; Martin et al., 2020; Saucet et al., 2021). Since TNL #1 proteins are found in the majority of eudicots and magnoliid stout camphor tree (*Cinnamomum micranthum*; Zhao et al., 2021) but not in conifers, *Amborella* or *Nymphaeales* (Figure 1B), this TIR group likely emerged in mesangiosperms before the split of monocots and eudicots and then was lost in monocots (Liu et al., 2021).

TNLs with the second taxonomically shared TIR nested in the NLR group called “NLR family 10” in Zhang et al. (2016). We refer to this NLR family 10-nested TNL group as “TNL #2” (Figure 1A). TNL #2 is shared by several species within two large groups of eudicots, the Rosids and Asterids. Our TIR phylogenetic analysis did not find evidence for this TIR group in Arabidopsis or *Amborella*. However, reciprocal BLASTP searches with the respective full-length TNL from domesticated tomato (*Solanum lycopersicum*; Solyc01g102920.2.1) suggest that these species have one putative orthologous sequence each (AT5G36930 in Arabidopsis). Because we define sequence groups based on TIR rather than NBARC, these Arabidopsis and *Amborella* TNLs do not fall into the TNL #2 group. In contrast to TNL #1 present in 1–4 copies per genome, the TNL #2 group expanded in some eudicot genomes (e.g. 54 genes in poplar *Populus trichocarpa*) (Figure 1B;Supplemental Figure S2B; iTOL link in the “Data availability section”; Zhang et al., 2016). It comprises ∼50% of the predicted TNLs in poplar, *Nb*, and domesticated tomato. We detected C-JID in TNL #2 (Supplemental Figure S2B; Figure 1A). Thus, TNL #1 and TNL #2 share the domain architecture including the C-terminal post-LRR region but differ in their taxonomic distribution and the number of copies per genome.

The third TIR group (we refer to as “conserved TIR-only”) corresponds to a small family of ∼200-aa-long proteins with a TIR-only architecture and 1–4 gene copies per genome. This group is present in 22 analyzed magnoliids, monocots, and eudicots but absent from conifers, *Amborella*, or *Nymphaeales* (Figure 1B), suggesting its emergence in mesangiosperms similar to the TNL #1 TIR. Strikingly, and in contrast to TNL #1, conserved TIR-only proteins are present in monocots. Arabidopsis TX3 and TX9 (Meyers et al., 2002; Nandety et al., 2013) fall into this TIR group. We noticed that the TIR-only protein RECOGNITION OF HOPBA1 (RBA1) does not belong to this conserved TIR-only group (Figure 1A; Nishimura et al., 2017). Therefore, we conclude that TIR-only protein domain architecture is not sufficient to assign TIR types.

The most taxonomically widespread plant TIR-containing proteins are TNPs (Figure 1B; Meyers et al., 2002; Sarris et al., 2016; Zhang et al., 2017a, 2017b; Lapin et al., 2022). TNPs are almost ubiquitous in analyzed species including the aquatic flowering plant duckweed watermeal (*Wolffia australiana*) with reduced NLR repertoire (Figure 1B;Supplemental Figure S4; Supplemental Files S10 and S11; Zhang et al., 2017a, 2017b; Baggs et al., 2020; Michael et al., 2020; Liu et al., 2021). The TNP group includes Arabidopsis TN17-like and TN21-like sequences (Nandety et al., 2013). Structure-guided comparison with plant NLRs revealed characteristic functional motifs in TNP NBARCs: Walker A (P-loop), RNBS-B with a TTR motif (Ma et al., 2020), Walker B, RNBS-C, GLPL, and MHD (Supplemental Figure S5). The TIR and NBARC sequences in TNPs are followed by C-terminal TPRs (Figure 1A; Lapin et al., 2022). Although fusions of nucleotide-binding domains with TPRs are common in fungi and bacteria (Dyrka et al., 2014; Gao et al., 2022; Lapin et al., 2022), TNP is the only TPR-containing class with an NLR-like architecture in plants. Custom HMM for the NBARC domain of plant TNPs (Supplemental File S12) and hmmsearch with Ensembl Genomes identified 1,680 hits most of which belong to plants (278), actinobacteria (427), and ascomycetes (793) (Potter et al., 2018). Multiple identified bacterial and fungal sequences have the TIR-NB-TPR or HET-NB-TPR architectures (Supplemental Figure S6A; Dyrka et al., 2014). Although BLAST searches for selected bacterial and fungal proteins identify Arabidopsis TNPs as primary hits, the similarity is based on the nucleotide-binding domains, not TIRs or TPRs. This is consistent with the TNP TIRs grouping away from bacterial TIRs (Toshchakov and Neuwald, 2020). NBARCs of TIR–NBARC–WD40 in red algae *Chondrus crispus* form a sister group to plant NBARC domains (Gao et al., 2018). Still, both reciprocal BLAST searches and phylogenetic grouping suggest that TIRs from *C. crispus* TIR–NBARC–WD40 sequences are not orthologous to TNP TIRs (Supplemental Figure S6B; Supplemental Files S13 and S14). Thus, plant TNPs show similarities to nonplant NLR-like proteins, but their evolutionary origin is unclear.

Taken together, the four TIR types we identify as shared by several taxonomic groups often have different protein domain architectures.

### A glutamate in the NADase catalytic motif is present in four taxonomically shared TIR groups

We assessed whether key residues critical for plant TIR functions are present in the four taxonomically shared TIR groups. The SH sequence motif is a part of the AE dimerization interface in TIRs of RESISTANT TO *PSEUDOMONAS SYRINGAE* 4 (RPS4) and other TNLs (Williams et al., 2014; Zhang et al., 2017a, 2017b; Ma et al., 2020; Martin et al., 2020; Lapin et al., 2022). This motif did not show a high level of sequence conservation across the four taxonomically shared TIR types (Figure 1C). A glycine residue that is necessary for TIR self-association via another interface and required for cell death and NADase activity of stiff brome (*Brachypodium distachyon*) *Bd*TIR and Arabidopsis RBA1 TIR-only proteins (Nishimura et al., 2017; Zhang et al., 2017a, 2017b; Wan et al., 2019) was conserved in the tested TIR groups except the TNPs (Figure 1C). AlphaFold2-predicted structures of selected conserved TIR-only proteins and TNP TIRs indicate that they differ from known plant TIRs at the TNL TIR-characteristic αD-helices (Supplemental Figure S7) (Bernoux et al., 2011; Lapin et al., 2022). The αD-helical region is important for cell death activities of TNL receptors RPS4 (Sohn et al., 2014) and L6 (Bernoux et al., 2011) and for 2′,3′-cAMP/cGMP synthetase activity found in several plant TIR domains (Yu et al., 2022). The glutamate residue which is indispensable for TIR NADase and 2′,3′-cAMP/cGMP synthetase activities (Essuman et al., 2018; Horsefield et al., 2019; Wan et al., 2019; Ma et al., 2020) was present in all four TIRs groups (Figure 1C;Supplemental Figure S7), pointing toward their probable catalytic activity.

### TIR groups show different co-occurrence patterns with ADR1, NRG1, and EDS1 family members

Since the EDS1 family connects plant TIR activity to resistance and cell death outputs (Lapin et al., 2020; Dongus and Parker, 2021; Lapin et al., 2022), we tested whether the distributions of EDS1 family members and the identified taxonomically shared TIR groups align across species. To infer numbers of putative EDS1, PAD4, and SAG101 orthologs per species, we built an ML tree for 200 sequences with an EP domain that uniquely defines the EDS1 family (Supplemental Figure S8; Supplemental Files S15 and S16; PFAM PF18117; Supplemental Table S3; Figure 1B). As expected, EDS1 and PAD4 were present in most seed plant species, while SAG101 was not detected in conifers, monocots, and *Caryophyllales* (Figure 1B;Supplemental Figure S8; Lapin et al., 2019; Baggs et al., 2020; Liu et al., 2021). Of the four taxonomically shared TIR groups, the conserved TIR-only type showed the highest co-occurrence with EDS1 and PAD4 in mesangiosperms (Figure 1B), indicating a possible functionally coevolved TIR-only-EDS1/PAD4 signaling module. In contrast, TNPs were present in nonseed land plants and aquatic plants that do not have the *EDS1* family genes (Figure 1BBaggs et al., 2020), pointing to EDS1 independent of TNP activities. Consistent with the co-occurrence of ADR1 and NRG1 NLRs with the EDS1 family (Collier et al., 2011; Lapin et al., 2019; Baggs et al., 2020), conserved TIR-only members distributed with ADR1s, whereas TNPs did not (Figure 1B;Supplemental Figure S9; Supplemental Files S17 and S18).

The above co-occurrence analyses confirmed that the TNL #1 group has a SAG101-independent distribution in angiosperms (Liu et al., 2021; Figure 1B). This prompted us to search for other protein orthogroups (OGs) that co-occur with TNL #1 and SAG101 (Supplemental Figure S10). Using Orthofinder, we built OGs for predicted protein sequences from 10 species. Five species (rice [*Oryza sativa*], pineapple [*Ananas comosus*], Norway spruce [*Picea abies*], *E. guttata*, columbine [*Aquilegia coerulea*]) lacked SAG101 and TNL #1 (Figure 1B; Zhang et al., 2016; Liu et al., 2021). One species (*A. hypochondriacus*) had TNL #1 but not SAG101. Finally, we included four species (Arabidopsis, *E. grandis*, poplar, domesticated tomato) with SAG101 and TNL #1. We imposed a strict co-occurrence pattern to retain only high-confidence candidates. Seven and five OGs followed the SAG101 and TNL #1 distribution, respectively. These findings were refined using reciprocal BLAST searches in genomes of the discriminatory species *B. vulgaris* (*TNL#1^+^/SAG101^−^*; Lapin et al., 2019; Liu et al., 2021), sesame (*Sesamum indicum*) and purple witchweed (*Striga hermonthica; TNL#1^−^*/*SAG101^−^*; Shao et al., 2016; Liu et al., 2021). After this filter, two OGs showed co-occurrence with SAG101—Arabidopsis hypothetical protein AT5G15190 and arabinogalactan AT2G23130/AT4G37450 (AGP17/AGP18) (Supplemental Figure S9). The other two OGs that co-occurred with the conserved angiosperm TNL #1 had Arabidopsis TERPENE SYNTHASE 4 (AT1G61120) and glutaredoxins ROXY16/17 (AT1G03020/AT3G62930) as representatives (Supplemental Figure S10). The functions of these genes in TIR-dependent defense are unknown. We concluded that conserved TIR groups show different distribution patterns in flowering plants and their co-occurrence with SAG101 is limited.

### Conserved *TIR-only* genes are transcriptionally induced in immune-triggered tissues

**Figure 2:**
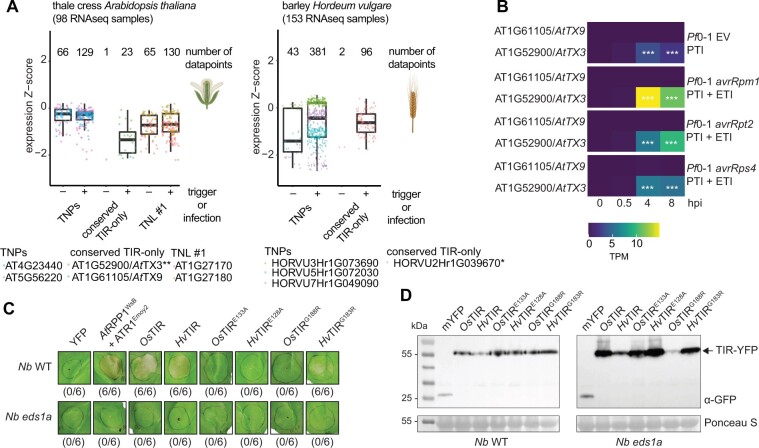
Conserved *TIR-only* genes are upregulated during immune signaling and their expression triggers *EDS1*-dependent cell death in *Nb*. A, Comparison of untriggered and immune-triggered expression of genes corresponding to taxonomically shared *TIR* groups in Arabidopsis and barley (*H. vulgare*). Data were taken from publicly available RNA-seq experiments (Supplemental Table S4) including immune-triggered and infected samples. The significance of the association between the expression of conserved *TIR-only* genes and the immune-triggered status of RNA-seq samples was assessed with Fisher’s exact test. The test evaluated whether the expression of conserved *TIR-only* genes (transcript per million >0) is more likely in immune-triggered samples. Asterisks next to names of the conserved *TIR-only* genes denote the significance level: *P < 0.05, **P < 0.01, ***P < 0.001. Minima and maxima of boxplots—first and third quartiles, respectively, center line—median, whiskers extend to the minimum and maximum values but not further than 1.5 interquartile range. Data points (number given above the boxplot) with the same color correspond to one gene. For details, check the “Data availability”. Created with elements from BioRender.com. B, Heatmaps showing expression of conserved *TIR-only* genes in Arabidopsis with PTI or ETI. Expression data were taken from Saile et al. (2020). Triggers include *P. fluorescens Pf*0-1 empty vector (EV) for PTI, *Pf*0-1 *avrRpm1*, *Pf*0-1 *avrRpt2*, and *Pf*0-1 *avrRps4* for PTI + ETI. Asterisks inside the heatmap indicate that conserved *TIR-only* AT1G52900 is upregulated at log_2_ fold change >4 and adjusted ***P < 0.001 relative to mock at 0-h postinfiltration (Saile et al., 2020). C, Macroscopic cell death symptoms induced by *Agrobacterium*-mediated overexpression of conserved monocot YFP-tagged TIR-only proteins in *Nb* WT and the *eds1a* mutant. Pictures were taken 3 days after agroinfiltrations. Numbers below panels indicate necrotic/total infiltrated spots observed in three independent experiments. D, TIR-only protein accumulation in infiltrated leaves shown in (C) was tested via immunoblot. Expected sizes for YFP-tagged TIR-only proteins and free YFP as control are indicated. All tested variants of conserved TIR-only proteins are expressed in *Nb* WT and *eds1a* lines. Ponceau S staining of the membrane served as loading control. The detection was performed for two independent experiments with similar results.

The broad taxonomic distribution of the four plant TIR groups prompted us to investigate their patterns of gene expression across plants. We used public RNA-seq data for seven plant species including Arabidopsis, *Nb*, barley (*Hordeum vulgare*), and *M. polymorpha* (referred to as *Marchantia*) (Supplemental Table S4 and Supplemental Figure S11; Figure 2A). The samples originated from infected- or immunity-triggered tissues as well as mock-treated or untreated controls. *TNP*, *TNL #1*, and *TNL #2* genes were expressed in both groups of RNA-seq samples from eudicots, monocots, and *Marchantia* (Supplemental Figure S11). Strikingly, the conserved *TIR-only* genes were either not detected or expressed at a very low level in nonstimulated tissues but they were expressed in immunity-triggered samples in both monocot and eudicot species (Figure 2A;Supplemental Figure S11; Meyers et al., 2002; Nandety et al., 2013). Fisher’s exact test for the association between the presence and absence of the immunity trigger and the expression (transcript per million [tpm] > 0) confirmed this pattern for conserved *TIR-only* transcripts in Arabidopsis, barley, and maize (*P* < 0.05; Figure 2A;Supplemental Figure S11). To explore further defense-related expression of *TIR*-*only* genes, we analyzed time series RNA-seq data from Arabidopsis with activated bacterial PAMP- or effector-triggered immune signaling (PTI and ETI; Figure 2B;Saile et al., 2020). Infiltration of leaves with the PTI-eliciting *Pseudomonas fluorescens Pf*0-1 containing a type III secretion system induced the conserved *TIR-only* gene *AtTX3*. *AtTX3* expression was also detected in samples with *Pf*0-1 delivering effectors recognized by NLRs (Figure 2B;Saile et al., 2020). Taken together, these observations suggest that the expression of the conserved *TIR-onl*y genes is responsive to immunity triggers in monocots and eudicots.

### Monocot-conserved TIR-only proteins induce *EDS1*-dependent cell death in *Nb*

Since the conserved TIR-only proteins co-occur with EDS1 and PAD4 (Figure 1B), we investigated if they trigger *EDS1*-dependent cell death similar to *B. distachyon* conserved TIR-only (*Bd*TIR) (Wan et al., 2019). For this, we cloned conserved *TIR*-only genes from rice (*Os*TIR, Os07G0566800) and barley (*Hv*TIR, HORVU2Hr1G039670) and expressed them as C-terminal mYFP fusions in *Nb* leaves using *Agrobacterium*-mediated transient expression assays (Figure 2C). Co-expression of RPP1^WsB^ with its matching effector ATR1^Emoy2^ as a positive control (Krasileva et al., 2010; Ma et al., 2020) resulted in cell death visible as leaf tissue collapse at 3 days post infiltration (dpi) (Figure 2C). mYFP as a negative control did not produce visible cell death symptoms (Figure 2C). Leaf areas expressing *Os*TIR or *Hv*TIR collapsed in *Nb* WT at 3 dpi but not in *eds1a* mutant leaves (Figure 2C). As the tested TIR-only proteins accumulated in *Nb eds1a* (Figure 2D), we concluded that monocot members of this TIR-only group induce *EDS1*-dependent cell death (Wan et al., 2019). The cell death response was fully suppressed when the catalytic glutamate residue was substituted by alanine (*Os*TIR^E133A^ and *Hv*TIR^E128A^; Figure 2C). Similarly, mutation of a conserved glycine at the BE TIR interface which is important for TIR NADase activity (Horsefield et al., 2019; Wan et al., 2019; Ma et al., 2020; Lapin et al., 2022) fully (*Os*TIR^G188R^) or partially (*Hv*TIR^G183R^) eliminated the cell death response (Figure 2C). All tested TIR-only mutant proteins accumulated in *Nb* WT and *eds1a* leaves (Figure 2D). These data show that monocot-conserved TIR-only proteins induce host cell death dependent on intact NADase catalytic sites and *EDS1* signaling.

### A maize clade IIa TNP induces *EDS1*-independent cell death in *N. tabacum*

**Figure 3:**
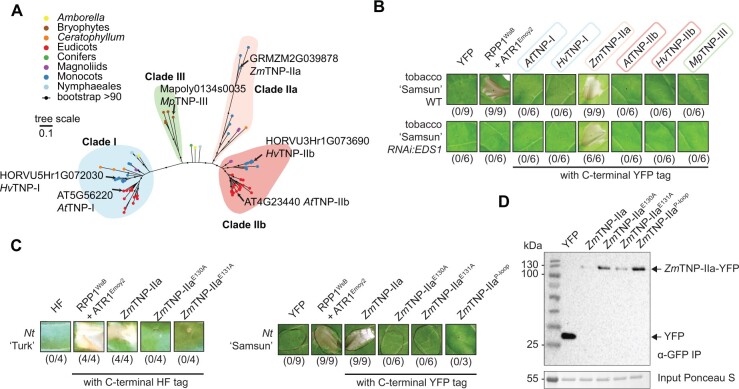
A maize TNP induces *EDS1*-independent cell death in *N. tabacum*. A, ML tree (from IQ-TREE, evolutionary model JTT+G4) of 77 predicted TNP NBARC (Supplemental File S12; hmmsearch E < 0.01) domains representing the plant species analyzed within this study. Branches with BS support ≥90% are marked with black dots. The three conserved TNP clades are highlighted with colored boxes. Clade nomenclature was partly adapted from Zhang et al. (2017). The scale bar is number of substitutions per site. B, Macroscopic cell death symptoms induced by *Agrobacterium*-mediated overexpression of C-terminally YFP-tagged TNP proteins from four major clades (A) in tobacco (*N. tabacum*) “Samsun” WT and the *RNAi:EDS1* knockdown line. Pictures were taken 5 days after agrobacteria infiltrations. Numbers below panels indicate necrotic/total infiltrated spots observed in three independent experiments. C, Overexpression of *Zm*TNP-IIa WT and mutant variants in the two adjacent putative catalytic glutamates (E130 and E131) or P-loop (G305A/K306A/T307A) in leaves of indicated tobacco varieties. Pictures were taken 5 days after agrobacteria infiltration. Numbers below panels indicate necrotic/total infiltrated spots observed in three independent experiments. D, *Zm*TNP-IIa-YFP protein accumulation in infiltrated leaves shown in (C) was tested via α-GFP IP and subsequent immunoblot. Expected sizes for YFP-tagged *Zm*TNP variants are indicated. Ponceau S staining of the IP input samples served as loading control. Similar results were obtained in another independent experiment.

TNPs persist in plant genomes regardless of the EDS1 family presence (Figure 1B;Supplemental Figures S2 and S4; Nandety et al., 2013; Zhang et al., 2017a, 2017b). We, therefore, hypothesized that TNPs function independently of *EDS1*. On the ML tree for TNP NBARC-like sequences selected with a custom-built HMM (Supplemental Files S12, S19, and S20), three major TNP clades were recovered, with one splitting into two subclades (Figure 3A). Clades I, IIa, and IIb match previously described TNP clades (Zhang et al., 2017a, 2017b). Clade IIa is missing from eudicots (Figure 3A; Zhang et al., 2017a, 2017b). All bryophyte TNP sequences formed a separate third clade (Clade III, Figure 3A). We selected representative sequences from the above three TNP clades to test whether they induce cell death: Arabidopsis AT5G56220 (*At*TNP-I, TN21) and barley HORVU5Hr1G072030 (*Hv*TNP-I) from Clade I, *Z. mays* GRMZM2G039878 from Clade IIa (*Zm*TNP-IIa), Arabidopsis AT4G23440 (*At*TNP-IIb, TN17), and barley HORVU3Hr1G073690 (*At*TNP-IIb) from Clade IIb, and *Marchantia* Mapoly0134s0035 from the bryophyte-specific Clade III (*Mp*TNP-III, Figure 3A). The C-terminally tagged TNPs (*Z. mays* TNP with 6xHis-3xFLAG [HF], others with mYFP) were expressed in leaves of tobacco (*N. tabacum*) “Samsun” or the corresponding *RNAi*:*EDS1* line (Duxbury et al., 2020) using *Agrobacterium*-mediated transient expression assays. We scored cell death visually as collapse of the infiltrated area at 5 dpi using co-expression of RPP1^WsB^-mYFP with effector ATR1^Emoy2^ as a positive control for *EDS1*-dependent cell death (Figure 3B). Expression of *Zm*TNP-IIa, but not other TNP forms, consistently elicited cell death which was *EDS1* independent (Figure 3B). None of the tested TNPs induced cell death in *Nb* leaves in our experiments. To test whether the predicted *Zm*TNP-IIa NADase catalytic glutamate is required for cell death, we substituted adjacent glutamate residues E130 or E131 in *Zm*TNP-IIa with alanines (*Zm*TNP-IIa^E130A^ and *Zm*TNP-IIa^E131A^; Figure 3C). Cell death was abolished for both mutant variants of YFP or HF-tagged *Zm*TNP-IIa in tobacco “Samsun” and “Turk.” *Zm*TNP-IIa-YFP cell death-inducing activity was also lost when the NBARC Walker A (P-loop) conferring ADP/ATP binding (Burdett et al., 2019) was mutated by replacing adjacent G305, K306, and T307 with alanines (*Zm*TNP-IIa^P-loop^; Figure 5). After purification with GFP-trap beads at 1 dpi before cell death symptoms were visible, all *Zm*TNP-IIa-YFP variants were detected by immunoblotting (Figure 3D). The cell death dependency on an intact P-loop suggests nucleotide-dependent activation of this TNP protein. We concluded that *Zm*TNP-IIa induces *EDS1*-independent cell death via its TIR NADase catalytic site and P-loop motif.

### *Botrytis*-infected *Nb tnp* mutants develop smaller necrotic lesions

**Figure 4:**
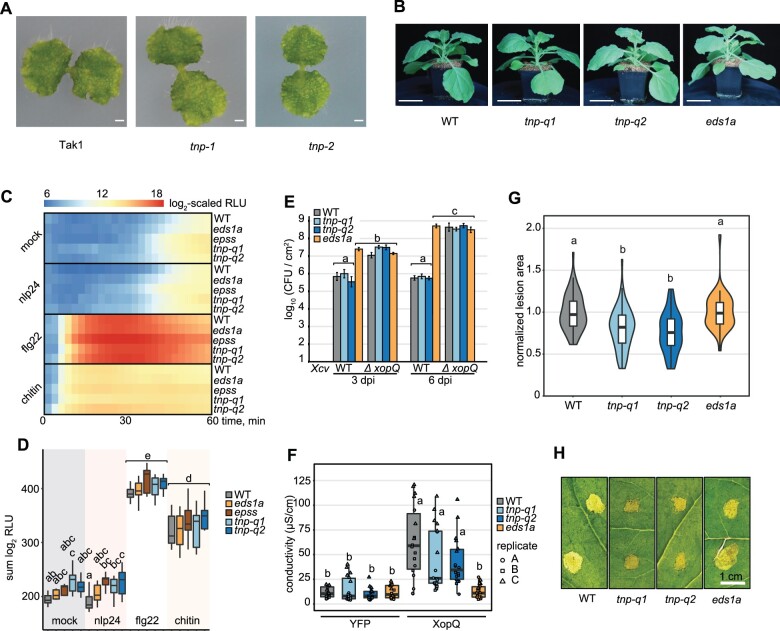
*TNPs* are not required for plant survival but negatively influence *B. cinerea* disease symptoms in *Nb*. A, Macroscopic images of 2-week-old *M. polymorpha* Tak1 WT and two independent *tnp* CRISPR knockout lines. Scale bars = 0.1 cm. Genomic sequences of the two *tnp* lines are depicted in Supplemental Figure S12. B, Side-view images of 4-week-old *Nb* WT, two independent *tnp* quadruple CRISPR knockout lines (*tnp-q1* and *tnp-q2*), and the *eds1a* mutant. Scale bars = 5.0 cm. Plants were grown in long-day (16-h light) conditions. Genomic sequences of the two *tnp* quadruple lines are depicted in Supplemental Figure S12. C, ROS burst upon several PAMP triggers in *Nb* WT, *eds1a*, *eds1a pad4 sag101a sag101b* (*epss*) and *tnp* quadruple mutants (*tnp-q1* and *tnp-q2*). Values are means of log_2_-transformed relative luminescence units (RLUs) after the addition of 2-μM nlp24, 200- nM flg22 or 4-mg mL^−1^ chitin and were recorded for 60 min, *n* = 10–12, from three independent biological replicates. D, Total ROS produced after 60 min PAMP treatment. Values are sums of log_2_-transformed RLU in (C). The letters above boxplots indicate significant differences among genotype-treatment combinations (Tukey’s HSD, *α* = 0.05, *n* = 10–12, from three independent biological replicates). E, *Xcv* growth assay in *Nb*. Plants were syringe infiltrated with *Xcv* 85-10 (WT) and *XopQ*-knockout strains (*Δ xopQ*) at OD_600_ = 0.0005. Bacterial titers were determined at 3 and 6 dpi. Genotype-treatment combinations sharing letters above boxplots are not significantly different (Tukey’s HSD, *α* = 0.01, *n* = 12, from three independent biological replicates). Error bars represent standard error. F, Electrolyte leakage assay as a measure of XopQ-triggered cell death in *Nb* 3 days after *Agrobacterium* infiltration (OD_600_ = 0.2) to express XopQ-Myc. YFP overexpression was used as negative control. Genotype-treatment combinations sharing letters above boxplots are not significantly different (Tukey’s HSD, *α* = 0.01, *n* = 18, from three independent biological replicates). G, Lesion area induced by *B. cinerea* strain B05.10 infection in *Nb*. Plants were drop-inoculated with spore suspension (5*10^5^ spores mL^−1^) and lesion areas were measured 48 h after inoculation. Values shown are lesion areas normalized to WT. Genotypes sharing letters above boxplots are not significantly different (Tukey’s HSD, *α* = 0.01, *n* = 10–12, from five independent biological replicates). Boxplot elements in (F) and (G): first and third quartiles define maximum and minimum, respectively, center line: median, whiskers extend to the minimum and maximum values but not further than 1.5 interquartile range. H, Macroscopic images of *B. cinerea*-induced lesions measured in (G).

To explore possible TNP functions, we developed two independent single and quadruple *tnp* mutants, respectively, in *Marchantia* and *Nb* (Supplemental Figure S12)*. Marchantia* has one *TNP* and *Nb* carries four *TNPs*. In both *Nb tnp* mutants and one *Marchantia tnp* mutant, the introduced mutations are predicted to cause frameshifts and stop codons before TIR in the predicted TNPs. One *Marchantia tnp* mutant has an in-frame deletion (Supplemental Figure S12). The tested *tnp* mutants displayed a similar morphology to respective WT (Figure 4, A and B). Hence, despite the high conservation and wide distribution in land plants, *TNP* genes are not essential for the vegetative growth of *Nb* or *Marchantia* under laboratory conditions.

Since PTI and TNL ETI readouts are well established for *Nb*, we used two independent *Nb tnp* mutant lines to assess whether *TNP* genes influence defense signaling. A reactive oxygen species (ROS) burst triggered by PAMPs flg22 or chitin was not altered in the *Nb tnp* mutants (Figure 4, C and D), indicating that *TNPs* are dispensable for PAMP perception and induction of immediate downstream ROS. Also, *Nb tnp* mutants supported WT-like growth of virulent *Xanthomonas campestris* pv. *vesicatoria* (*Xcv*) bacteria without a XopQ effector triggering TNL Roq1 (*Xcv ΔxopQ*Figure 4E). In TNL Roq1 ETI *Xcv* growth assays, the *tnp* mutants were also indistinguishable from resistant WT plants, although the *eds1a* mutant was susceptible to *Xcv* (Figure 4E; Adlung et al., 2016; Schultink et al., 2017). Similarly, Roq1-induced cell death was unaffected in the *tnp* mutants after *Agrobacterium*-mediated transient expression of XopQ (Figure 4F), whereas *eds1a* displayed low electrolyte leakage similar to the negative control (Figure 4F). Therefore, *TNP*s are likely dispensable for the tested PTI and ETI outputs in *Nb.*

We analyzed the responses of the *Nb tnp* mutants to infection by the necrotrophic fungus *Botrytis cinerea*. Both *tnp* lines developed smaller necrotic lesions 48 h after spore application while the *eds1a* mutant behaved like WT (Figure 4, G and H). The phenotypes of WT and *eds1a* compared to *tnp* mutants when challenged with *B. cinerea* suggest that *Nb TNP*s, directly or indirectly, contribute to *B. cinerea* lesion development via an *EDS1*-independent mechanism.

## Discussion

TIR signaling domains mediate cell death and immune responses across kingdoms, including plants (Essuman et al., 2022; Lapin et al., 2022). Here, we analyzed plant TIR conservation and distribution using recently available genomes from major lineages of land plants and ML phylogenetic tools (Nguyen et al., 2015; Chernomor et al., 2016). We recovered four taxonomically shared plant TIR groups which so far have no described functions in defense signaling. While two of these TIR groups matched conserved TIR-only and TNPs (Meyers et al., 2002; Nandety et al., 2013; Zhang et al., 2017a, 2017b; Toshchakov and Neuwald, 2020; Lapin et al., 2022), two other TIR groups are from angiosperm TNL families (Zhang et al., 2017a, 2017b; Liu et al., 2021) (Figure 1A). Consistent with differing patterns of co-occurrence with the EDS1 family (Figure 1B), conserved monocot TIR-only proteins and a maize TNP triggered cell death dependently and independently of *EDS1*, respectively (Figures 2 and 3). Thus, variation exists in the *EDS1* dependency of plant TIR-promoted cell death.

Although TNL NBARCs of land plants are nested within NBARCs of charophytes (Gao et al., 2018), none of the four conserved TIR groups included sequences from unicellular chlorophyte algae (Supplemental Figure S2), red algae *C. crispus*, or charophyte *Klebsormidium nitens* (Supplemental Figure S6). Also, our reciprocal BLAST searches did not find putative TNP orthologs in charophytes *K. nitens* and *Chara braunii*. Hence, the four taxonomically shared TIR groups probably evolved in land plants. A better coverage of algal diversity with phylogenomic information will help to clarify the origin and evolution of plant TIRs.

TNPs, the most conserved TIR protein architecture in land plants (Figure 1B;Supplemental Figure S4; Meyers et al., 2002; Zhang et al., 2017a, 2017b), are also present in bacteria and fungi (Supplemental Figure S6A; Dyrka et al., 2014; Gao et al., 2022). Notably, bacterial NLR-like proteins with TPRs activate cell death after sensing phage proteins via the C-terminal TPRs and forming tetramers resembling plant TNL resistosomes (Ma et al., 2020; Martin et al., 2020; Gao et al., 2022). We anticipate that the initial functional characterization of *Zm*TNP-IIa presented here (Figure 3) will prompt further analysis of the roles for TPR-containing NLR-like proteins across kingdoms.

We show that the full-length protein domain architecture is insufficient to define TIR groups. Conserved TIR-only proteins are phylogenetically distinct from Arabidopsis TIR-only RBA1 (also known as *At*TX1), *At*TX12 (Nandety et al., 2013; Nishimura et al., 2017), and *At*TX0 (Yu et al., 2022) which are closer to TIRs of TNLs RPS4 and LAZARUS 5 (Supplemental Figure S2). TIR-only proteins from both conserved TIR-only and RBA1-like groups can trigger *EDS1*-dependent cell death and are transcriptionally induced in response to immunity triggers (Figure 2;Supplemental Figure S11; Nandety et al., 2013; Nishimura et al., 2017; Wan et al., 2019; Lapin et al., 2022; Yu et al., 2022). The similar physiological properties of evolutionarily distinct TIR-only proteins suggest functional conservation of TIR-only groups in plant immunity (Yu et al., 2022). Indeed, both conserved TIR-only proteins *Bd*TIR and RBA1 promoted EDS1–SAG101–NRG1A complex formation, indicating their capacity to produce the same or similar EDS1 pathway-inducing nucleotide signals for immunity (Huang et al., 2022; Jia et al., 2022). Since TIR-only is the most widespread TIR protein architecture in green plants (Figure 1B;Supplemental Figure S1; Sun et al., 2014), comparative analyses of different TIR-only groups will be crucial to understand how plant immunity networks operate.

We found differences in copy number of the different TIR group proteins, with several dozens of TNL #2 in some eudicot genomes and 1–4 genes of other TIR groups (Figure 1B;Supplemental Table S3). NLRs show high copy number variation in plants (Baggs et al., 2017), ranging from 3,400 NLRs in bread wheat (*Triticum aestivum*) (Steuernagel et al., 2020) to one in *W. australiana* (Michael et al., 2020). High variability in copy number is often associated with the generation of diversity and recognition specificity in a sensor (Nozawa and Nei, 2008; Kanduri et al., 2013; Prigozhin and Krasileva, 2021). The presence of the effector-sensing C-JID domain in multiple TNL #2 further suggests they act as pathogen sensors (Figure 1A; Dodds et al., 2001; Ma et al., 2020; Martin et al., 2020). It remains to be determined whether and how sensor TNLs connect functionally with conserved TIR-only groups in the immune system, although it is possible that the transcriptionally induced *TIR*-only genes serve as defense potentiators downstream of TNLs and other pathogen stress detection systems (Pruitt et al., 2021; Tian et al., 2021; Lapin et al., 2022; Parker et al., 2022; Yu et al., 2022).

The absence of conserved TNL #1 and TIR-only clades in several plant species (Figure 1B) suggests that these TIR protein families are not essential for plant viability. TNPs are almost ubiquitous in land plants (Figure 1B; Zhang et al., 2017a, 2017b) and we generated mutants of all *TNPs* in *Marchantia* and *Nb*. *Nb tnp* mutants and the effectively *TIR*-less *Marchantia tnp* mutant were viable and had no obvious developmental defects under laboratory conditions (Figure 4). Thus, TNPs and other TIR-containing proteins are not essential for plant development in contrast to Toll and TLR signaling in animals (Anthoney et al., 2018).

We found that conserved TIR-only proteins from monocots and *Zm*TNP-IIa triggered cell death in *Nb* or tobacco leaves (Figures 2, C, 3, B and C) and this required a glutamic acid residue in their predicted catalytic motifs (Figure 1C). These findings align with the conserved glutamate being important for cell death triggering enzymatic activities of TIR domains (Essuman et al., 2018; Horsefield et al., 2019; Wan et al., 2019; Lapin et al., 2022; Yu et al., 2022). Notably, expression of *Zm*TNP-IIa produced cell death in the tobacco *RNAi:EDS1* line (Figure 3B) as did SARM1 and HopAM1 in an *Nb eds1* mutant (Horsefield et al., 2019; Eastman et al., 2021). Consistent with HopAM1-producing *EDS1*-independent cell death (Eastman et al., 2021), this bacterial TIR effector did not trigger complex formation between EDS1–PAD4 and ADR1-L1 (Huang et al., 2022). Based on these earlier findings and the observations that the *RNAi:EDS1* line did not show TNL-dependent effector-triggered cell death (Figure 3B; Duxbury et al., 2020), we conclude that *Zm*TNP-IIa can induce *EDS1*-independent cell death in contrast to all other so far studied plant TIR proteins (Lapin et al., 2022). EDS1 heterodimers selectively react to TIR domain enzymatic products for cell death and resistance (Dongus et al., 2022; Huang et al., 2022; Jia et al., 2022). Consistent with this, the 2′,3′-cAMP/cGMP synthetase activity of TIR-only protein RBA1 was dispensable for complex formation between EDS1–SAG101 dimers and NRG1A (Huang et al., 2022; Yu et al., 2022). Hence, different requirements of plant TIR proteins for EDS1 in the promotion of cell death that we report here might reflect in part their varying enzymatic capacities and preferences.

## Materials and methods

### Prediction, alignment, and phylogenetic analysis of TIRs and other domains

Proteomes of 39 plant species (Supplemental Table S1) were screened for TIR domains using hmmsearch (HMMER 3.1b2, –incE 0.01) with TIR and TIR-related HMMs from the Pfam database (Supplemental Table S2). Redundant TIR sequences found with different TIR and TIR-like HMMs and showing overlap >20 amino acids were removed. The minimal domain length for TIRs was set to 50 amino acids. For NBARC domain, the minimal length was set at 150 amino acids. Multiple sequence alignments (MSAs) were constructed with MAFFT (version 7.407, fftns or ginsi, with up to 1,000 iterations) (Katoh et al., 2002). MSAs were filtered and columns with ˃40% gaps were removed in the Wasabi MSA browser (http://was.bi/). The ML phylogenetic trees were inferred with IQ-TREE (version 1.6.12, options: -nt AUTO -alrt 1,000 -bb 1,000 -bnni; options for the EDS1 family tree: -nt AUTO -b 500; Nguyen et al., 2015; Chernomor et al., 2016). Their visualization and annotation were performed using iTOL version 5 (Letunic and Bork, 2021) or the R package ggtree (Yu, 2020). Sequence data were processed in R with the Biostrings package (https://bioconductor.org/packages/Biostrings). Prediction of other domains was performed with hmmsearch (HMMER 3.1b2, –E 0.01) on Pfam A from release 34.0.

### Presence and absence analysis of proteins consistent with SAG101 and conserved angiosperm TNL #1

Orthofinder (version 2.3.11) was run on the following proteomes: P.abies 1.0, Osativa 323 v7.0, Acomosus 321 v3, Acoerulea 322 v3, Ahypochondriacus 459 v2.1, Slycopersicum 514 ITAG3.2, Mguttatus 256 v2.0, Athaliana 167 TAIR10, Egrandis 297 v2.0, and Ptrichocarpa 533 v4.1. Norway spruce (*Picea abies*) proteome was downloaded from congenie.org, all other proteomes were downloaded as the latest version of the primary transcript from the Phytozome database (version 12) on 31 March 2020. Then, we extracted OGs that followed the pattern of presence and absence of interest using the following custom scripts extract_orthogroup_TNL_absent_v2.py and extract_orthogroup_SAG101_absent_v2.py. Scripts and orthofinder output are available on github (https://github.com/krasileva-group/TIR-1_signal_pathway.git). Arabidopsis (*A. thaliana*) genes from each OG were searched using tBLASTn against sesame (*S. indicum*) (Ensembl Plants), purple witchweed (*S. hermonthica*) (COGE), and sugar beet (*B. vulgaris*) (Ensembl Plants). The top hit was then searched with BLASTX or BLASTP (if a gene model was available) back against the Arabidopsis proteome.

### Determining numbers of ADR1 and NRG1 sequences

The number of ADR1 and NRG1 homologs was determined by constructing an ML tree for NBARC sequences in all species under study (PF00931.22, E < 0.001). NBARCs ADR1 and NRG1 form readily distinguishable sister groups (Shao et al., 2016). The derived counts for previously analyzed species were compared with earlier reports (Baggs et al., 2017; Lapin et al., 2019). For rice (*O. sativa*) and barley (*H. vulgare*), ADR1 sequences were missed by NBARC HMM. For flooded gum (*E. grandis*), multiple NRG1 sequences were missed by the HMM search. They were later recovered with reciprocal BLASTP searches. The ADR1/NRG1 counts based on the HMM could differ from the inferences based on the full-length sequence searches.

### Generation of expression vectors

TNP coding sequences without Stop codons were amplified from cDNA (Arabidopsis Col-0, barley “Golden Promise,” rice “Kitaake,” common liverwort (*M. polymorpha*, accession Tak1) using oligonucleotides for TOPO or BP cloning (Supplemental Table S5). Coding sequences were amplified with Phusion (NEB, Ipswich, MA, USA) or PrimeStar HS (Takara Bio, Shiga, Japan) polymerases and cloned into pENTR/D-TOPO (Thermo Fisher Scientific, Waltham, MA, USA) or pDONR221 vectors and verified by Sanger sequencing. Mutations in the sequences were introduced by side-directed mutagenesis using specific oligonucleotides (Supplemental Table S5). Recombination of sequences into pXCSG-GW-mYFP (Witte et al., 2004) expression vector was performed using LR Clonase II enzyme mix (Life Technologies, Carlsbad, CA, USA). Correct insertion was tested by restriction enzyme digests. *ZmTNP-IIa* was synthesized by TWIST bioscience with codon optimization for expression in *Nb*, two fragments were required to synthesize maize (*Z. mays*) *ZmTNP-IIa*. The two fragments were ligated during golden gate cloning into pICSL22011 (Supplemental Table S5) using BsaI restriction sites. Vectors were verified by Sanger sequencing. Site-directed mutagenesis of *Zm*TNP-IIa was carried out using Agilent technologies QuickChange Lightning Site-Directed Mutagenesis Kit (210518) (oligonucleotides listed in Supplemental Table S5). Expression vectors harboring RPP1^WsB^ and ATR1^Emoy2^ were previously published (Ma et al., 2020).

### Transient protein expression and cell death assays in *Nicotiana* species

*Agrobacterium tumefaciens* strains GV3101 pMP90RK or pMP90 with plasmids of interest were infiltrated into *Nb* or tobacco (*N. tabacum*) leaves at a final OD_600_ of 0.5. For *Nb* infiltrations, *A. tumefaciens* strain C58C1 pCH32 with the viral DNA silencing repressor P19 was added (OD_600_ = 0.1). Before infiltration using a needle-less syringe, *A. tumefaciens* strains were incubated in induction buffer (10-mM MES pH 5.6, 10-mM MgCl_2_, 150-nM acetosyringone) for 1–2 h in the dark at room temperature. Protein samples were collected at 2 dpi for immunoblot assays. Macroscopic cell death was recorded using a camera at 3 dpi. For electrolyte leakage assays, six 8-mm leaf disks were harvested for infiltrated leaf parts at 3 dpi and washed in double-distilled water for 30 min. After washing, leaf disks were transferred into 24-well plates, each well filled with 1-mL ddH_2_O. The conductivity of water was then measured using a Horiba Twin ModelB-173 conductometer at 0 and 6 h.

### Protein enrichment via IP

To enrich YFP-tagged proteins transiently expressed in tobacco leaves, immunoprecipitation (IP) was performed. For this, four 1-cm leaf disks were harvested per sample at 1 dpi and ground in liquid nitrogen. About 1.5 mL of extraction buffer (10% (v/v) glycerol, 100-mM Tris–HCl pH 7.5, 5-mM MgCl_2_, 300-mM NaCl, 10-mM DTT, 0.5 IGEPAL CA-630, 1× plant protease inhibitors, 2% (w/v) poly(vinylpolypyrrolidone)) were added and tubes inverted at 4°C for 10 min. The dissolved samples were centrifuged at 4,500*g* at 4°C for 35 min. The supernatant was passed through Miracloth (Merck, Kenilworth, NJ, USA; 475855) and a 50-µL input sample was taken, mixed with 50-µL Lämmli buffer, and boiled at 95°C for 10 min. The remaining sample was mixed with 20-µL GFP Trap agarose bead slurry (Proteintech, gta) and incubated with inverting at 4°C for 2 h. Afterward, tubes were centrifuged at 500*g*, 4°C for 1 min to pellet the GFP trap beads. Supernatant was removed and the beads resuspended in 1-mL IP-buffer (10% (v/v) glycerol, 100-mM Tris–HCl pH 7.5, 5-mM MgCl_2_, 300-mM NaCl, 0.5% IGEPAL CA-630, 1× plant protease inhibitors; Merck; 11873580001). Beads were washed 3 times with IP-buffer, centrifuging at 500*g*, 4°C for 1 min each time to pellet the beads. After the last centrifugation, the supernatant was removed, 50-µL Lämmli buffer was added, and the samples boiled at 95°C for 10 min.

### Immunoblot analysis

To test protein accumulation in *Nb* plants, three 8-mm leaf disks were harvested per sample at 2 dpi and ground in liquid nitrogen. Ground tissue was dissolved in 8-M urea buffer, vortexed for 10 min at RT, and centrifuged at 16,000*g* for 10 min (Ma et al., 2020). Total protein extracts were resolved on a 10% sodium dodecyl sulfate polyacrylamide gel and subsequently transferred onto a nitrocellulose membrane using the wet transfer method. Tagged proteins in total protein or after affinity purification (see above) were detected using α-GFP antibodies (Merck; 11814460001) in a 1:5,000 dilution (1× TBST, 2% milk (w/v), 0.01% (w/v) NaAz), followed by incubation with HRP-conjugated secondary antibodies (Merck; A9044). Signal was detected by incubation of the membrane with Clarity and Clarity Max substrates (BioRad, Hercules, CA, USA; 1705061 and 1705062) using a ChemiDoc (BioRad). Membranes were stained with Ponceau S for loading control (Merck; 09276-6X1EA-F).

### ROS burst assays in *Nb*

A ROS burst in response to elicitors was measured according to Bisceglia et al. (2015). Leaf disks of 4 mm from fourth or fifth leaves of 5-week-old *Nb* plants were washed in double-distilled (mQ) water for 2 h and incubated in 200 μL of mQ water in 96-well plates (Greiner Bio-One; #655075) under aluminum foil overnight. The mQ was then substituted by a solution of L-012 (Merck SML2236, final 180 μM) and horseradish peroxidase (Merck; P8125-5KU, 0.125 units per reaction). Elicitors flg22 (Genscript; RP19986, final 0.2 μM), chitin (from shrimp shells, Merck C7170, resuspended in mQ for 2 h and passed through 22 μm filter, final 4 mg mL^−1^), and nlp24 (Genscript, synthesized peptide from *Hyaloperonospora arabidopsidis* Necrosis and ethylene-inducing peptide 1-like protein 3 AIMYAWYFPKDSPMLLMGHRHDWE, crude peptide, final 2 μM) were each added to a 250-μL reaction. Luminescence was recorded on a Glomax instrument (Promega, Madison, WI, USA) at 2.5 min intervals. Log_2_-transformed relative luminescence units were integrated across time points for the statistical analysis (ANOVA, Tukey’s HSD test).

### *Xcv* infection assays in *Nb*

*Xcv* bacteria were infiltrated in 4-week-old *Nb* mutant leaves at a final OD_600_ of 0.0005. The *Xcv* strain carrying the type III effector XopQ (WT) and one strain lacking XopQ (Δ *xopQ*) were dissolved in 10-mM MgCl_2_. Bacterial solutions were infiltrated using a needleless syringe. After infiltration, plants were placed in a long-day chamber (16-h light/8-h dark at 25°C/23°C). Three 8-mm leaf disks representing technical replicates were collected at 0, 3, and 6 dpi. To isolate the bacteria, discs were incubated in 1 mL 10-mM MgCl_2_ supplemented with 0.01% Silwet (v/v) for 1 h at 28°C at 600 rpm shaking. Dilutions were plated on NYGA plates containing 100-mg L^−1^ rifampicin and 150 mg L^−1^ streptomycin.

### *Botrytis* infection assays in *Nb*

*Botrytis cinerea* strain B05.10 was grown on potato glucose agar medium for 20 days before spore collection. Leaves from 4- to 5-week-old soil-grown *Nb* were drop inoculated by placing 10 μL of a suspension of 5 × 10^5^ conidiospores mL^−1^ in potato glucose broth medium on each side of the middle vein (4/6 drops per leaf). Infected plants were placed in trays at room temperature in the dark. High humidity was maintained by covering the trays with a plastic lid after pouring a thin layer of warm water. Under these experimental conditions, most inoculations resulted in rapidly expanding water-soaked necrotic lesions of comparable diameter. Lesion areas were measured 48-h postinfection by using ImageJ.

### Generation of *M. polymorpha tnp* CRISPR/Cas9 mutants

Guide RNA design was performed using CRISPR-P 2.0 (http://crispr.hzau.edu.cn/CRISPR2/) where the sequence of Mapoly0134s0035 was used as an input (guide RNAs are listed in Supplemental Table S5). *Marchantia polymorpha* Tak-1 was transformed as described in Kubota et al. (2013) with the exception that *A. tumefaciens* strain GV3101 pMP90 was used. Briefly, apical parts of thalli grown on 1/2 Gamborgs B5 medium for 14 days under continuous light were removed using a sterile scalpel, and the basal part of each thallus was sliced into four parts of equal size. These fragments were then transferred to 1/2 Gamborgs B5 containing 1% (w/v) sucrose under continuous light for 3 days to induce calli formation before co-culture with *A. tumefaciens*. On the day of co-culture, *A. tumefaciens* grown for 2 days in 5-mL liquid LB with appropriate antibiotics at 28°C and 250 rpm were inoculated in 5-mL liquid M51C containing 100-µM acetosyringone at an estimated OD_600_ of 0.3–0.5 for 2.5–6 h in the same conditions. The regenerated thalli were transferred to sterile flasks containing 45-mL liquid M51C, and *A. tumefaciens* was added at a final OD_600_ of 0.02 in a final volume of 50 mL of medium with 100-µM acetosyringone. After 3 days of co-culture agitated at 400 rpm under continuous light, the thalli fragments were washed 5 times with sterile water and then incubated 30 min at room temperature in sterile water containing 1-mg mL^−1^ cefotaxime to kill bacteria. Finally, plants were transferred to 1/2 Gamborgs B5 containing 100-µg mL^−1^ hygromycin and 1-mg mL^−1^ cefotaxime and grown under continuous light for 2–4 weeks. Successful mutagenesis was validated by polymerase chain reaction amplification (oligonucleotides listed in Supplemental Table S5) and subsequent Sanger sequencing. Two independent lines were selected for further experiments.

### Generation of *Nb tnp* CRISPR/Cas9 mutants

Guide RNA design was performed using CRISPR-P 2.0 (http://crispr.hzau.edu.cn/CRISPR2/) where the four *NbTNP* sequences were inputted (guide RNAs are listed in Supplemental Table S5). *Nb* WT plants were transformed according to (Ordon et al., 2019). Successful mutagenesis was validated by PCR amplification (oligonucleotides listed in Supplemental Table S5) and subsequent Sanger sequencing. Two homozygous quadruple mutants were selected. *Nb* WT line used as a background for transformation was included in all experiments with the *tnp* mutants as a control.

### Analysis of publicly available immune-related RNA-seq datasets

RNA-seq data (Supplemental Table S4) were downloaded from Sequence Read Archive with sra toolkit (SRA Toolkit Development Team, https://github.com/ncbi/sra-tools; version 2.10.0). After FastQC quality controls (Andrews, S. 2010; A Quality Control Tool for High Throughput Sequence Data; http://www.bioinformatics.babraham.ac.uk/projects/fastqc/), reads were trimmed with Trimmomatic (version 0.38, LEADING:5 TRAILING:5 SLIDINGWINDOW:4:15 MAXINFO:50:0.8 MINLEN:36) (Bolger et al., 2014). Transcript abundance was quantified with Salmon (version 1.4.0, –fldMean = 150 –fldSD = 20 for single-end reads, –validateMappings –gcBias for paired-end reads) (Patro et al., 2017). The tximport library (version 1.22.0) was used to get the gene expression level in tpm units (Soneson et al., 2015). Since RNA-seq samples are coming from diverse studies that use different library preparation methods and sequencing platforms, tpm values were standardized per sample and the derived *z*-scores were used for visualization of the expression levels. Genome versions used as a reference for transcript quantification: Arabidopsis—TAIR10, rice—IRGSP-1.0, barley—IBSCv2, maize—B73v4, *Marchantia*—v3.1, and *Nb*—NbD (Kourelis et al., 2019). *NLR* genes were predicted with NLR-Annotator (https://github.com/steuernb/NLR-Annotator; Steuernagel et al., 2020). To test for the association of a gene expression with immune-triggered status of tissue, Fisher’s exact test for contingency tables was applied followed by Bonferroni correction for multiple testing.

### Data availability

Scripts for the gene expression analysis and extraction of the TIR domains can be found in Zenodo (10.5281/zenodo.7005015). Annotated phylogenetic trees are accessible via iTOL (https://itol.embl.de/shared/lapin).

### Accession numbers

Sequence data from this article can be found in the GenBank/EMBL and Solgenomics data libraries under accession numbers: *Bd*TIR—XP_003560074.3, *Os*TIR—Os07G0566800, *Hv*TIR—XP_044965689.1, *Zm*TNP-IIa—AQK58421.1, *Mp*TNP-III—PTQ29824.1, *Nb*TNPs (Solgenomics)—Niben101Scf08517g00007.1 (NbD annotation—NbD042327.1), Niben101Scf11738g00026.1 (NbD047748.1), Niben101Scf04988g02021.1 (NbD031432.1), and Niben101Scf10074g00009.1 (NbD045462.1).

## Supplemental data

The following materials are available in the online version of this article.

**Supplemental Figure S1.** TIR distribution across 39 plant species.

**Supplemental Figure S2.** Complete TIR phylogeny across tested plant species.

**Supplemental Figure S3.** Phylogeny of TIR-associated NBARC domains.

**Supplemental Figure S4.** TNP NBARC ML phylogenetic tree including sequences from aquatic plants.

**Supplemental Figure S5.** TNP NBARC sequence alignment and motifs.

**Supplemental Figure S6.** Similarity of plant TNPs to nonplant proteins.

**Supplemental Figure S7.** Alignment of AlphaFold2-predicted structures of conserved TIR-only and TNP TIRs against solved structures of TIRs from TNL proteins.

**Supplemental Figure S8.** EP domain phylogeny to access presence/absence of EDS1 components in plant proteomes.

**Supplemental Figure S9.** NBARC domain phylogeny for plant species used in the study.

**Supplemental Figure S10.** Presence and absence of TNL #1, SAG101 and OGs co-occurring with them across selected seed plant species.

**Supplemental Figure S11.** *TIR* gene expression in immune-triggered tissues.

**Supplemental Figure S12.** Mutant alleles of *M. polymorpha* and *Nb tnp* lines.

**Supplemental Table S1.** List of species used in this study.

**Supplemental Table S2.** List of HMMs used in this study.

**Supplemental Table S3.** Counts of EDS1 family members across species.

**Supplemental Table S4.** List of RNA-seq accessions.

**Supplemental Table S5.** Oligonucleotides used in this study.

**Supplemental File S1.** Alignment used to produce ML tree in Supplemental Figure S2a.

**Supplemental File S2.** ML tree in Supplemental Figure S2a (Newick format).

**Supplemental File S3.** Alignment used to produce ML tree in Supplemental Figure S2b.

**Supplemental File S4.** ML tree in Supplemental Figure S2b (Newick format).

**Supplemental File S5.** Protein sequences containing TIR domains in Supplemental Figure S2a.

**Supplemental File S6.** Alignment used to produce ML tree in Figure 1a.

**Supplemental File S7.** ML tree in Figure 1a (Newick format).

**Supplemental File S8.** Alignment used to produce ML tree in Supplemental Figure S3.

**Supplemental File S9.** ML tree in Supplemental Figure S3 (Newick format).

**Supplemental File S10.** Alignment used to produce ML tree in Supplemental Figure S4.

**Supplemental File S11.** ML tree in Supplemental Figure S4 (Newick format).

**Supplemental File S12**. Custom HMM based on TNP NBARC.

**Supplemental File S13.** Alignment used to produce ML tree in Supplemental Figure S6b.

**Supplemental File S14.** ML tree in Supplemental Figure S6b (Newick format).

**Supplemental File S15.** Alignment used to produce ML tree in Supplemental Figure S8.

**Supplemental File S16.** ML tree in Supplemental Figure S8 (Newick format).

**Supplemental File S17.** Alignment used to produce ML tree in Supplemental Figure S9.

**Supplemental File S18.** ML tree in Supplemental Figure S9 (Newick format).

**Supplemental File S19.** Alignment used to produce ML tree in Figure 3a.

**Supplemental File S20.** ML tree in Figure 3a (Newick format).

## Supplementary Material

kiac480_Supplementary_Data

## Notes

D.L., O.J., E.L.B., K.V.K., and J.E.P. conceived the project. D.L., O.J., and E.L.B. performed sequence and phylogenetic analyses. D.L., O.J., and K.K. analyzed RNA-seq data. O.J., J.A.D., D.L., C.U., K.M., and H.N. developed CRISPR/Cas9 mutant lines. O.J., H.L.L., F.L., and E.L.B. performed the cell death and pathogen assays. O.J., E.L.B., D.L., K.V.K., and J.E.P. analyzed the data. O.J., D.L., and J.E.P. wrote the manuscript with contributions from E.L.B. and K.V.K.

The author responsible for the distribution of materials integral to the findings presented in this article in accordance with the policy described in the Instructions for Authors (https://academic.oup.com/plphys/pages/general-instructions) is Dmitry Lapin (d.lapin@uu.nl).
