# Supplementary material for: Variation in plant Toll/Interleukin-1 receptor domain protein dependence on ENHANCED DISEASE SUSCEPTIBILITY 1
Source: Plant Physiol. 2022 Oct 13;191(1):626–42. doi: 10.1093/plphys/kiac480 (PMC9806590; doi:10.1093/plphys/kiac480)
Supplement: kiac480_Supplementary_Data [file kiac480_supplementary_data.zip › kiac480_Supplementary_Data/SupplementalFigures.pdf]

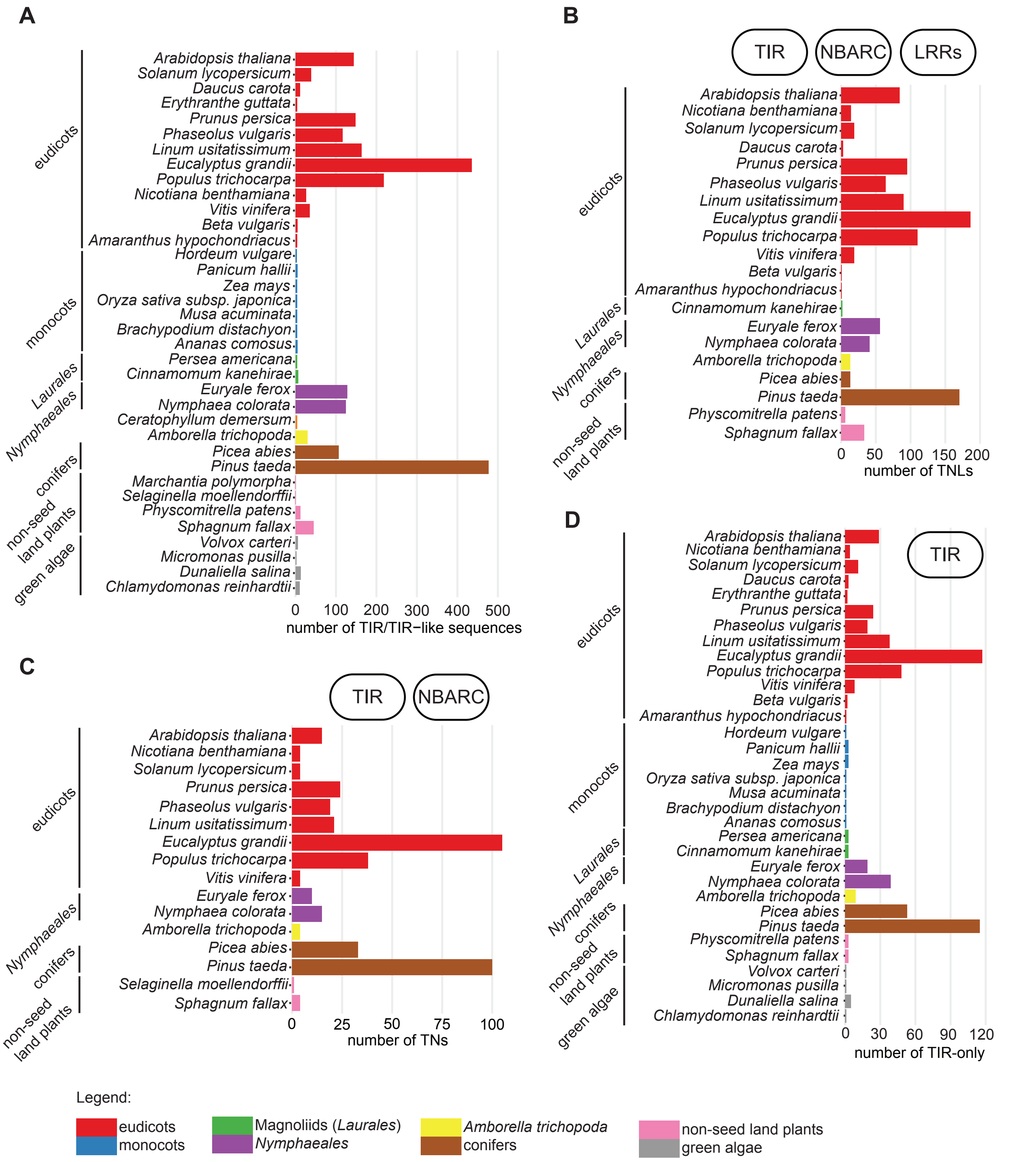


**Supplemental Figure S1. TIR distribution across 39 plant species.**
**(A)** Total number of TIR domains predicted in plant species representing major algae and land plant taxa. **(B)** Number of proteins with TIR, nucleotide-binding domain shared by APAF-1, certain *R*-gene products and CED-4 (NBARC) and leucine-rich repeat (LRR) domains (TNL). **(C)** Number of proteins with TIR and NBARC domains (TN). **(D)** Number of proteins with a TIR-only architecture (<400 amino acid (aa) long sequences with no other predicted domains).

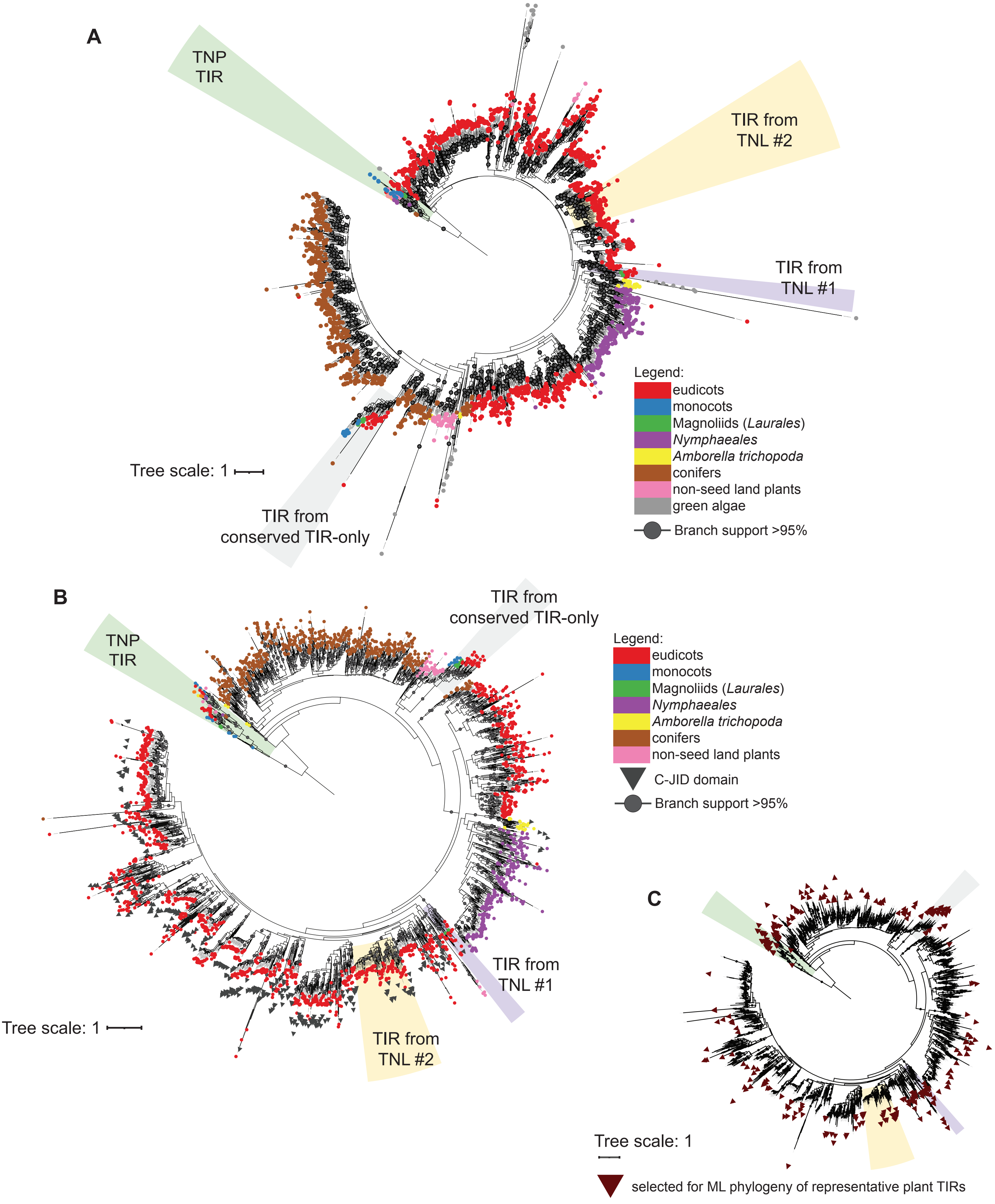
A
TNP TIR
TIR from TNL #2
TIR from TNL #1
TIR from conserved TIR-only
Tree scale: 1
Legend:
eudicots
monocots
Magnoliids (Laurales)
Nymphaeales
Amborella trichopoda
conifers
non-seed land plants
green algae
Branch support >95%
B
TNP TIR
TIR from conserved TIR-only
Legend:
eudicots
monocots
Magnoliids (Laurales)
Nymphaeales
Amborella trichopoda
conifers
non-seed land plants
C-JID domain
Branch support >95%
TIR from TNL #1
TIR from TNL #2
Tree scale: 1
C
Tree scale: 1
selected for ML phylogeny of representative plant TIRs

**Supplemental Figure S2. Complete TIR phylogeny across tested plant species.**
**(A)** Maximum likelihood (ML) tree (from IQ-TREE, evolutionary model JTT+F+R10) of 2348 predicted TIR domain sequences from 39 plant species (including green algae). Branches with BS support ≥95% are marked with black dots. Conserved groups with TIRs from more than one taxonomic order are highlighted with colored boxes. **(B)** ML tree (from IQ-TREE, evolutionary model JTT+F+R9) for 2317 predicted TIR domain sequences (same dataset as in A but excluding algal TIRs). Branches with BS support ≥95% are marked with black dots. Conserved groups with TIRs from more than one order are highlighted with colored boxes. **(C)** Same tree as in B with red triangles marking position of selected TIR sequences used to construct ML tree in Figure 1a. The scale bars correspond to number of substitutions per site.

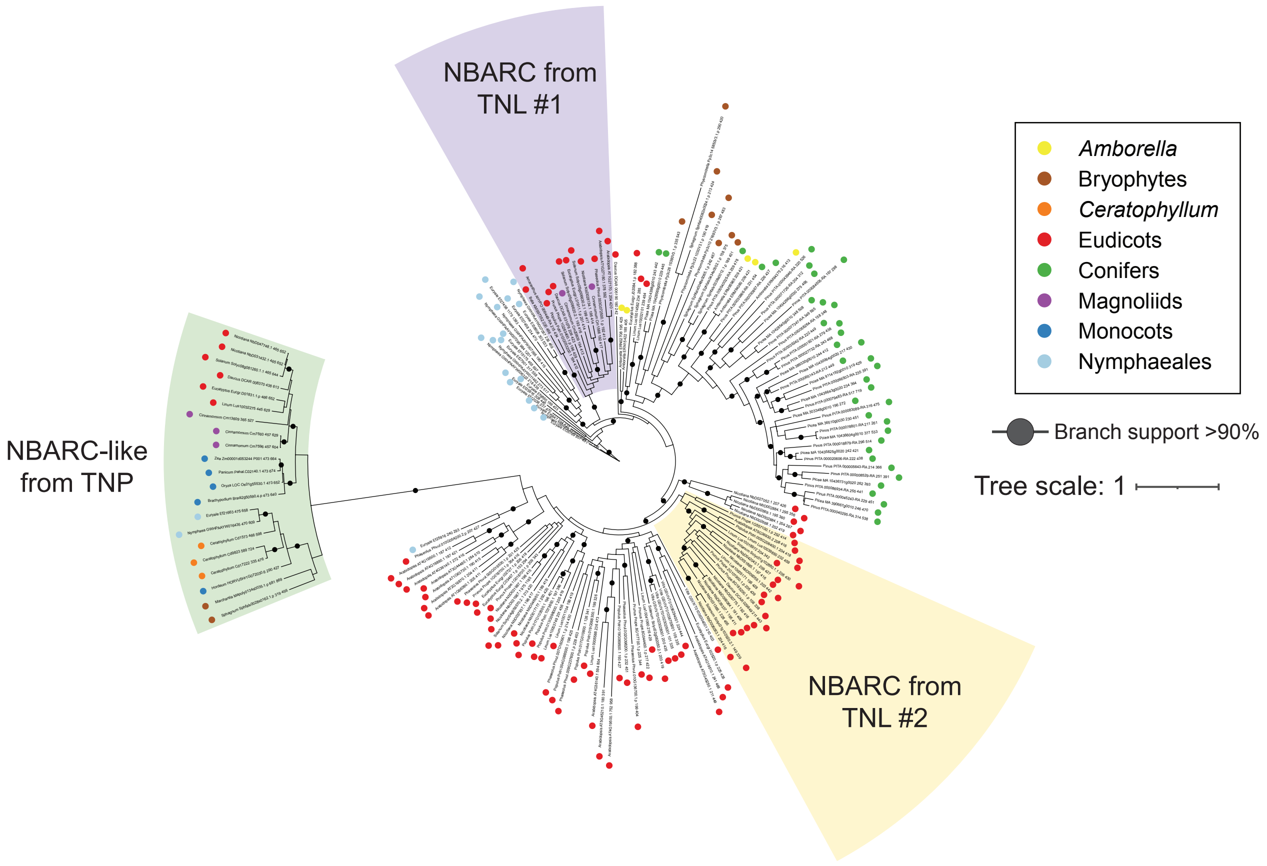


**Supplemental Figure S3. Phylogeny of TIR-associated NBARC domains.**
ML tree (from IQ-TREE, evolutionary model JTT+F+R5) for 178 NBARC domain sequences predicted as additional domains in the representative TIR protein dataset shown on the ML tree in Figure 1a. Branches with BS support ≥90% are marked with black dots. Conserved groups with TIRs from more than one species are highlighted with colored boxes. The scale bar corresponds to number of substitutions per site.

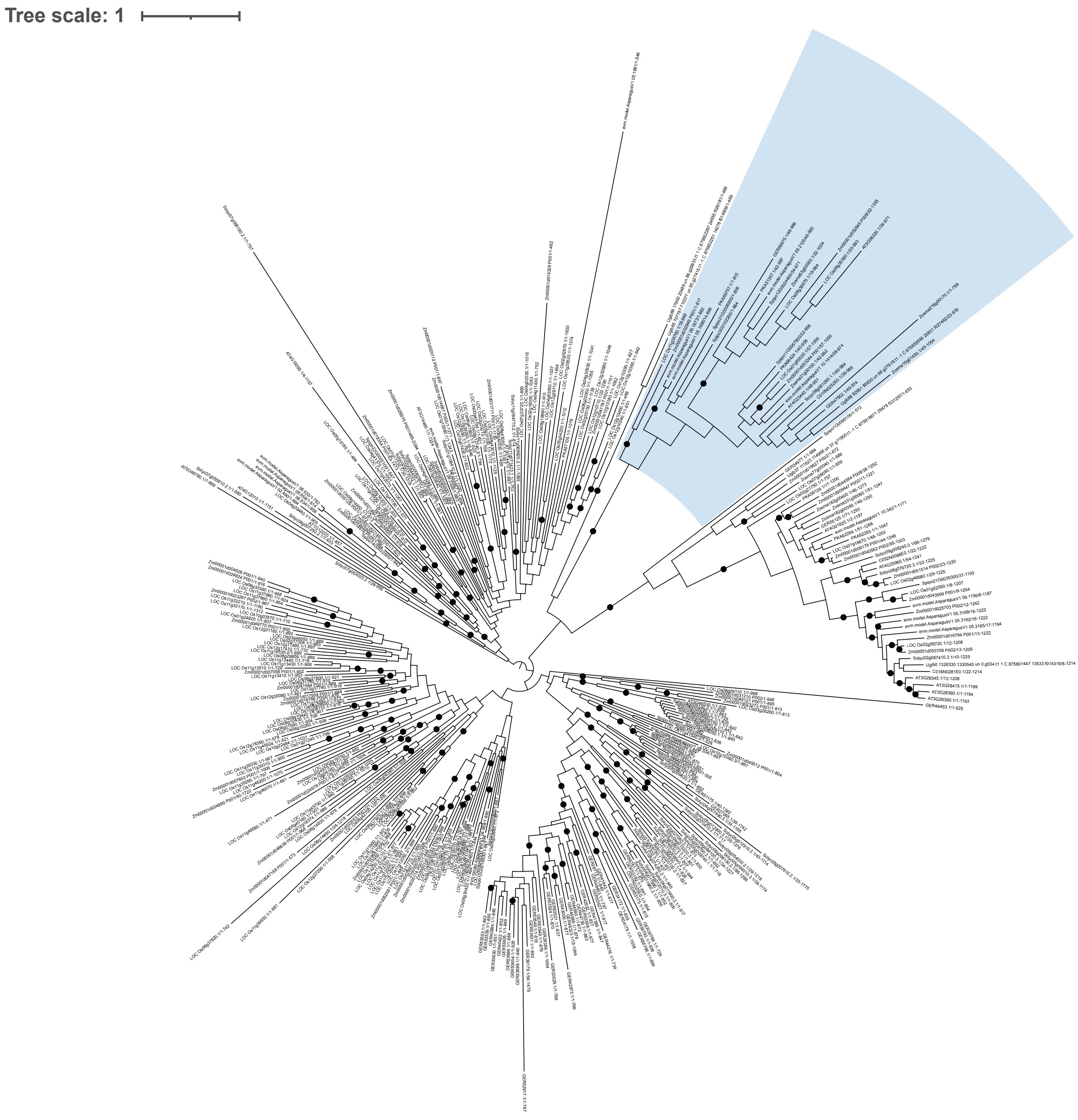


**Supplemental Figure S4. TNP NBARC ML phylogenetic tree including sequences from aquatic plants.**

ML tree (from RAxMLv8.2.9, evolutionary model PROTCATJTT) for 201 NBARC domain selected with hidden Markov model for TNP NBARC (hmmsearch at -E 0.01). Tree includes species with and without EDS1. Branches with bootstrap support ≥90% are marked with black dots. The blue clade indicates TNP containing proteins. The scale bar is number of substitutions per site.

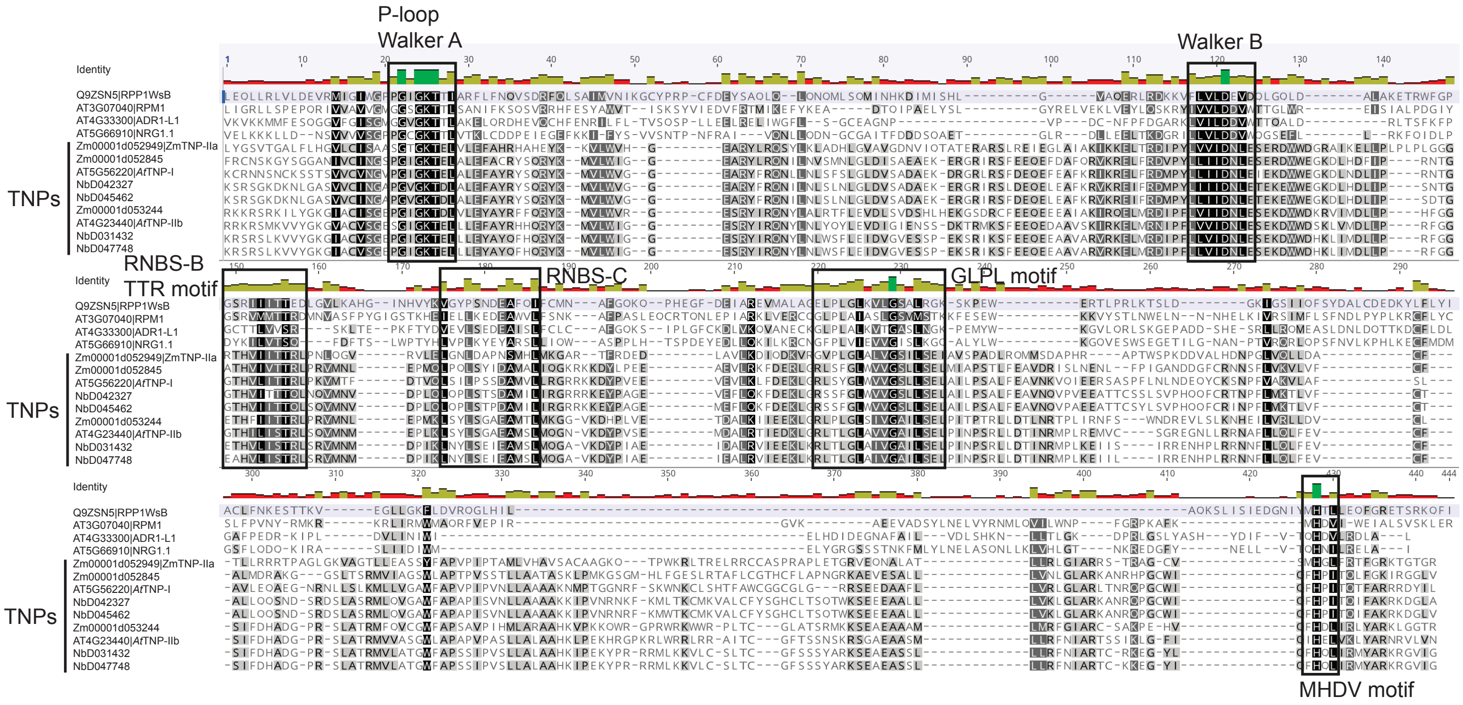


**Supplemental Figure S5. TNP NBARC sequence alignment and motifs.**
Amino acid sequence alignment (from MUSCLE) of NBARC domains from NLR and TNP proteins. Black boxes highlight conserved motifs. Sites with >90% gaps are hidden to help the visualization.

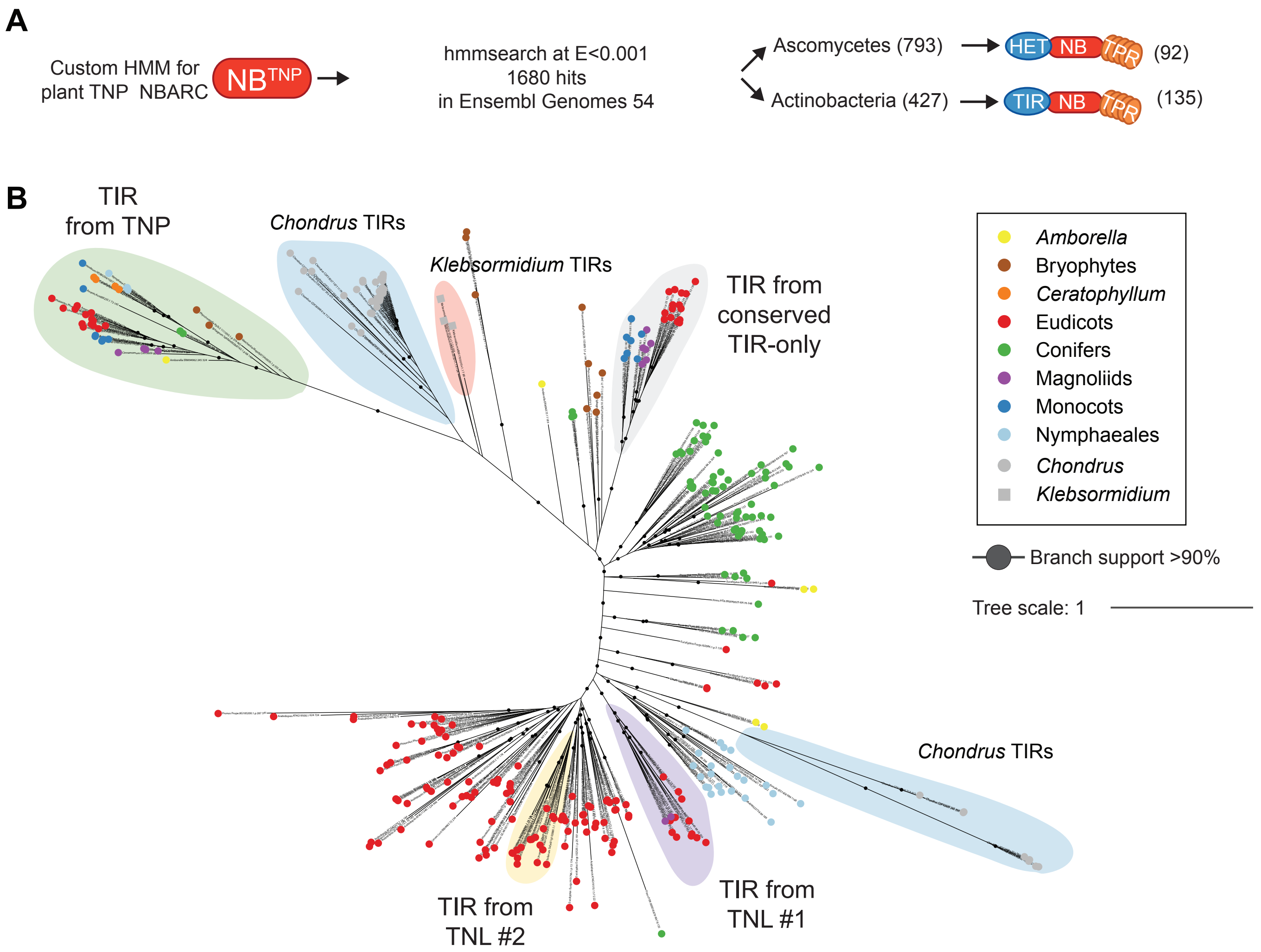


**Supplemental Figure S6. Similarity of plant TNPs to non-plant proteins.**
**(A)** HMM for plant TNP NBARC domain identifies similarities in bacteria and fungi. Multiple hits have TIR-NB-TPR and HET-NB-TPR domain organization. The search was performed with EBI hmmsearch on Ensembl Genomes (release 54) at -E 0.001 --domE 0.001 --incE 0.001 --incdomE 0.001 (https://www.ebi.ac.uk/Tools/hmmer/search/hmmsearch). **(B)** ML tree (from IQ-TREE, evolutionary model WAG+F+R7) for 353 predicted TIR domain sequences (same dataset as in Figure1A but including predicted TIRs from the red algae *Chondrus crispus* and the charophyte *Klebsormidium nitens*). Branches with BS support ≥90% are marked with black dots. Conserved groups with TIRs from more than one species and *Chondrus*- and *Klebsormidium*-specific groups are highlighted with colored boxes. The scale bar corresponds to number of substitutions per site.

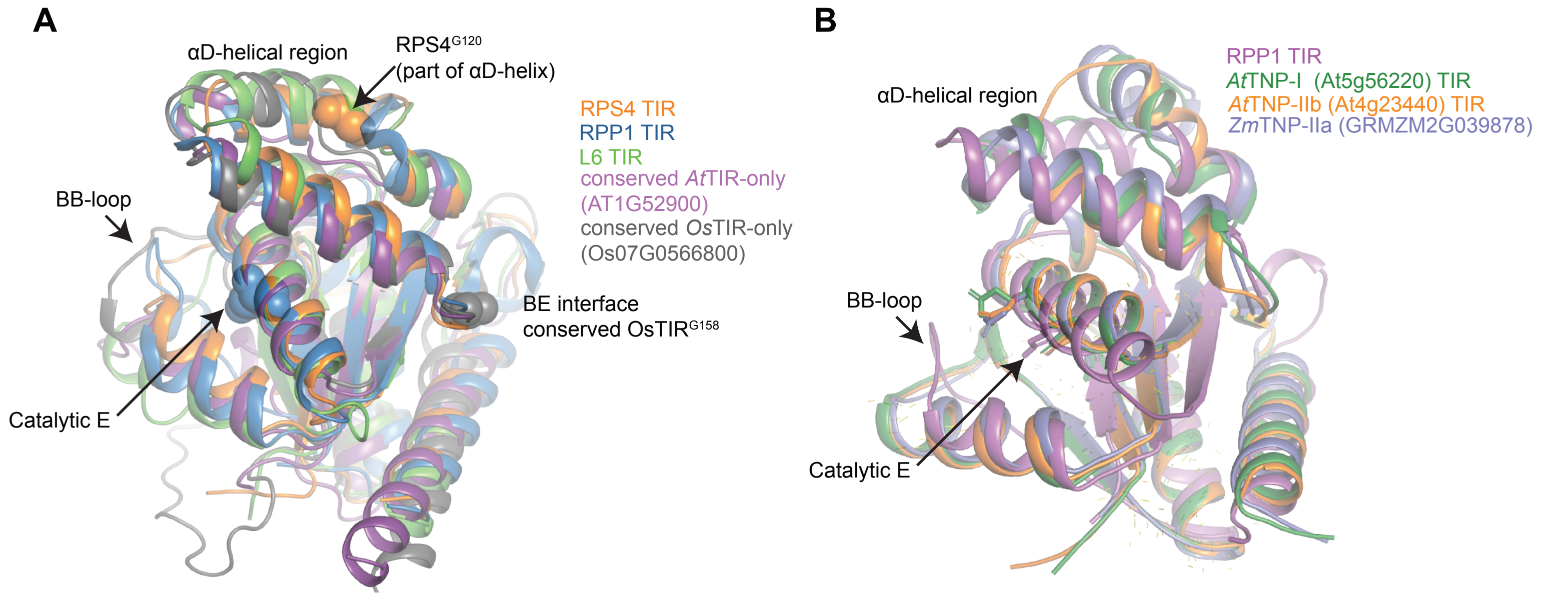


**Supplemental Figure S7. Alignment of AlphaFold2-predicted structures of conserved TIR-only and TNP TIRs against solved structures of TIRs from TNL proteins.**
**(A)** Solved structures of the RPS4 (PDB:4c6t, chain B), RPP1$^{WsB}$ (PDB:7crc, chain C) and L6 (PDB:3ozi, chain A) TIR domains were aligned in PyMol (v3.7) to predicted structures of conserved TIR-only proteins from Arabidopsis (AT1G52900, AF-Q9C931-F1) and rice (Os07G0566800, AF-Q7XIJ6-F1). Positions of major TIR-TIR AE and BE self-association interfaces as well as the BB-loop region and catalytic glutamates are shown with arrows. αD helical region of conserved TIR-only proteins AT1G52900 and Os07G0566800 is likely less structured compared to RPS4, RPP1 and L6 TIRs. **(B)** Alignment of predicted TIR structures of *Arabidopsis* (AT5G56220 - AF-Q9FH17-F1; AT4G23440 - AF-O81740-F1) and *Zea mays* (GRMZM2G039878 - AF-K7U4T9-F1) TNPs to TIR of RPP1$^{WsB}$ (PDB:7crc, chain C). Tested TNP TIRs likely differ from RPP1$^{WsB}$ TIR in the β-strand D and the αD helical region.

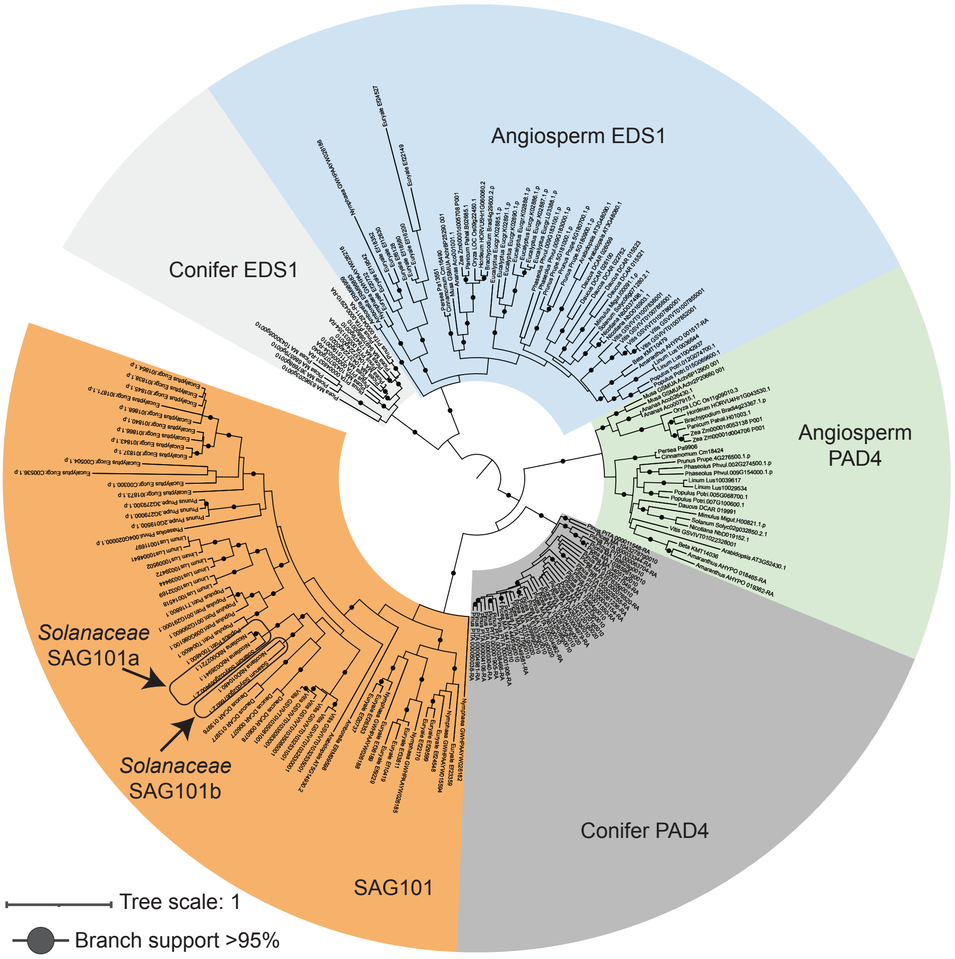


**Supplemental Figure S8. EP domain phylogeny to access presence/absence of EDS1 components in plant proteomes.**
ML tree (from IQ-TREE, evolutionary model JTT+F+R7) for predicted EP domain sequences. Based on phylogeny, numbers of putative EDS1, PAD4 and SAG101 orthologues were calculated per species. Branches with BS support ≥95% are marked with black dots. Conserved groups with EP domains from EDS1, PAD4 or SAG101 are highlighted with colored boxes. The scale bar is number of substitutions per site.

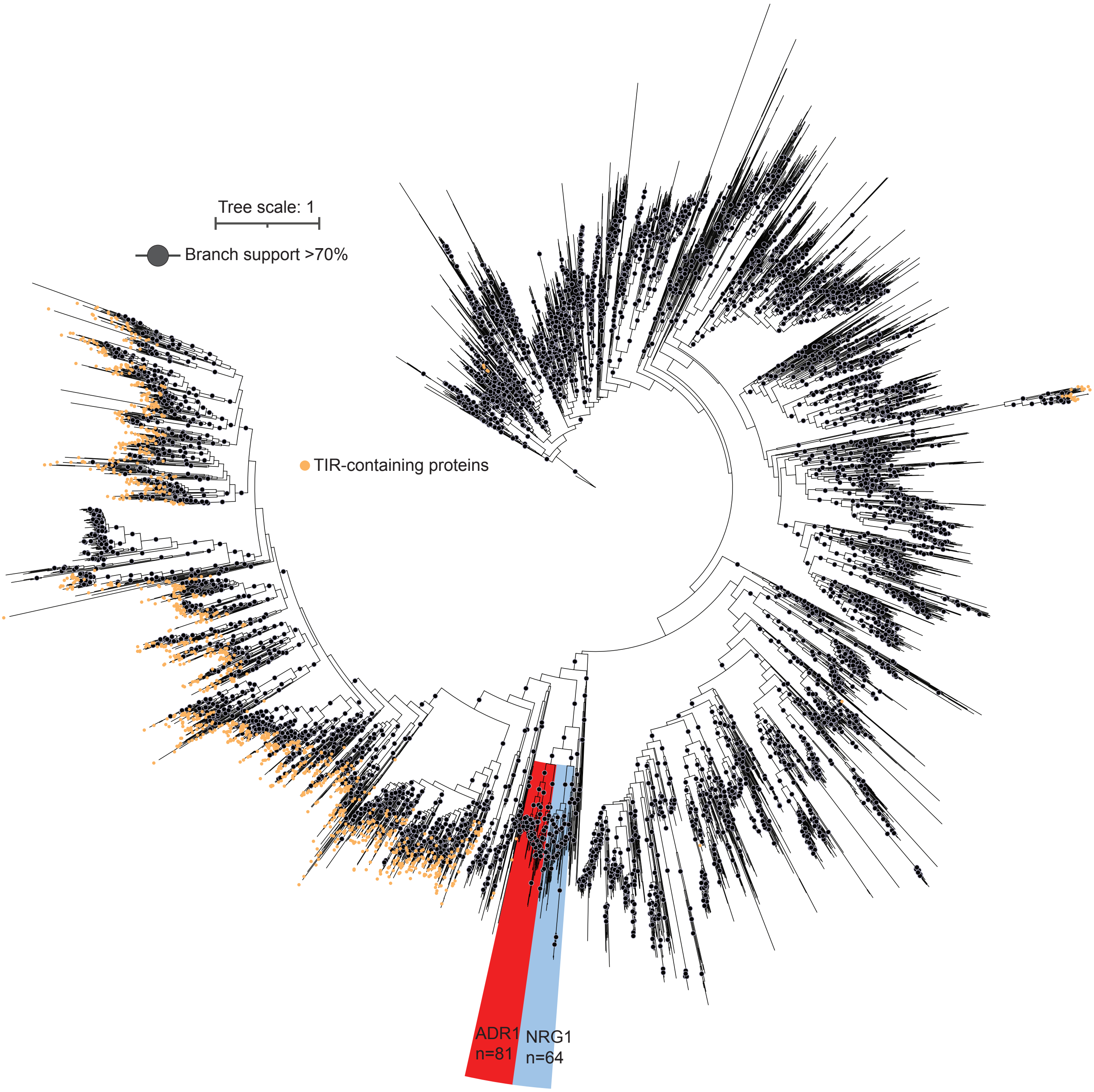


**Supplemental Figure S9. NBARC domain phylogeny for plant species used in the study.**
ML tree (from IQ-TREE, evolutionary model JTTDCMut+F+R7) for predicted 6161 NBARC sequences from species used in this study. Based on phylogeny, numbers of predicted ADR1 (red) and NRG1 (blue) orthologues was calculated per species (Supplemental Table S3). Branches with BS support ≥70are marked with black dots. NBARCs from TIR-containing full-lenth proteins are annotated with the orange dot. The tree is unrooted but it is shown as rooted to aid visualization. The scale bar corresponds to number of substitutions per site.

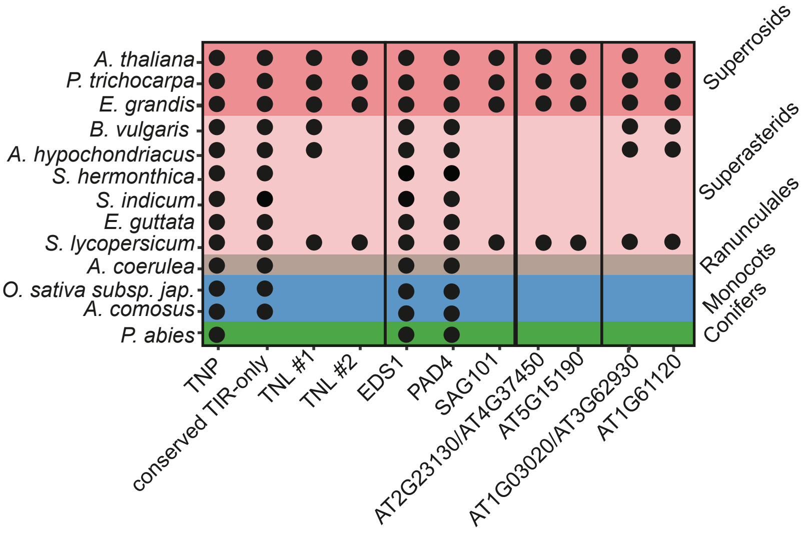


**Supplemental Figure S10. Presence-absence of TNL #1, SAG101 and orthogroups co-occurring with them across selected seed plant species.**

Dot plot to indicate co-occurrence of protein families. Presence of TNP, TIR-only and TNLs is based on phylogenetic analysis in Figure 1. For all other columns a black dot indicates presence of a protein belonging to that protein orthogroup as identified by Orthofinder, BLASTP or reciprocal tBLASTn.

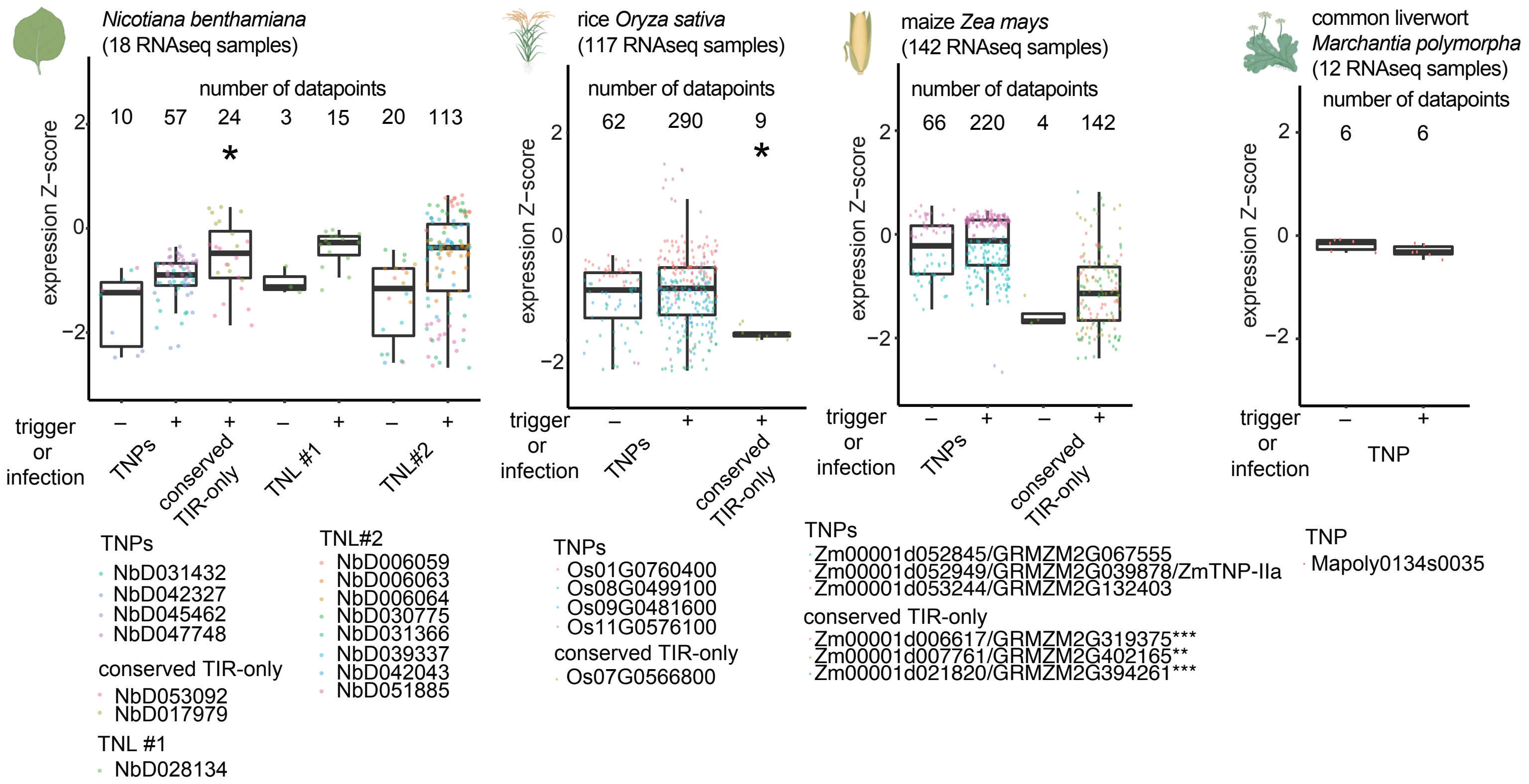


**Supplemental Figure S11.** ***TIR*** **gene expression in immune-triggered tissues.**
Comparison of untriggered and immune-triggered expression of NLRs and genes corresponding to conserved TIR groups in *Nicotiana benthamiana*, rice (*Oryza sativa*), maize (*Zea mays*) and the common liverwort Marchantia polymorpha. Data were taken from publicly available RNAseq experiments (Supplemental Table S4) including immune-triggered and infected samples. Star above the boxplot indicates that expression of the genes is not detected in untriggered samples. The significance of association between the expression of conserved TIR-only genes and the immune-triggered status of RNAseq samples was assessed with Fisher's exact test. The test evaluated whether the expression of conserved TIR-only genes (transcript per million > 0) is more likely to be detected in the immune-triggered samples. Asterisks next to the name of the conserved TIR-only genes denote the significance level from the Fisher's exact test: * $p<0.05$, ** $p<0.01$, *** $p<0.001$. Minima and maxima of boxplots - first and third quartiles, respectively, center line - median, whiskers extend to the minimum and maximum values but not further than 1.5 interquartile range. Datapoints (number given above the boxplot) with the same color correspond to one gene. For details, check the Data availability section. Created with elements from BioRender.com.

**A**

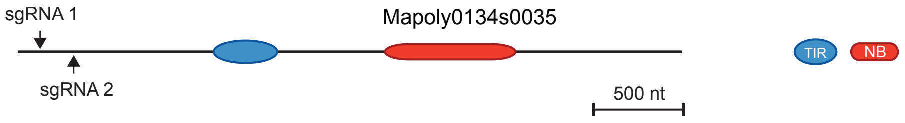


Mapoly0134s0035 sgRNA 1

WT: GCTCTCTCGTCGTCCTCGTCGTCGTCGCTCGCGTGCCC
*tnp1*: GCTCTCTCGTCGTCCTCGT-GTCGTCGCTCGCGTGCCC
frameshift at aa 42, stop after aa 404
*tnp2*: GCTCTCTCGTCGTCCTCGTCGTCGTCGCTCGCGTGCCC

Mapoly0134s0035 sgRNA 2

WT: CGCCCTCGTCGCCCTCCG---------TCTCTCACAGCATTGC
*tnp1*: CGCCCTCGTCGCCCTCCG---------TCTCTCACAGCATTGC
*tnp2*: CGCCCTCGTCGCCCTCCTCGCGTCGTCTCTCTCACAGCATTGC
three aa insertion RRL after S102

**B**

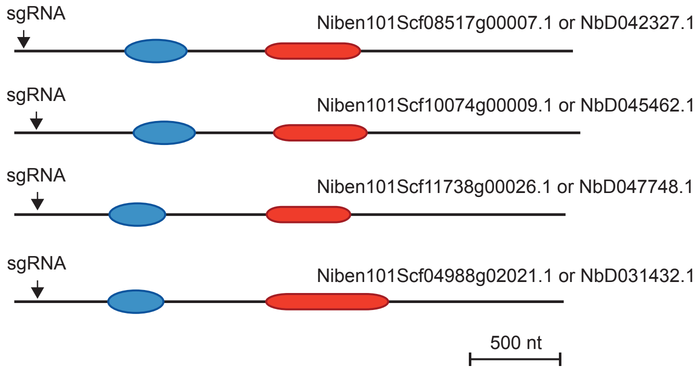


Niben101Scf08517g00007.1 or NbD042327.1

WT: CCACTGTTCTTCCTTCAGG-TCAGCAGAAATTGGTAGC
*tnp-q1*: CCACTGTTCTTCCTTCAGGGTCAGCAGAAATTGGTAGC
frameshift leading to stop after aa 56
*tnp-q2*: CCACTGTTCTTCCT------TCAGCAGAAATTGGTAGC
frameshift leading to stop after aa 54

Niben101Scf10074g00009.1 or NbD045462.1

WT: CCACAGTTCTTCCTTCA-GGTCAGCAGAAATTGGTAGC
*tnp-q1*: CCACAGTTCTTCCTTCATGGTCAGCAGAAATTGGTAGC
frameshift leading to stop after aa 56
*tnp-q2*: CCACAGTTCTTCCTTCA--GTCAGCAGAAATTGGTAGC
frameshift leading to stop after aa 46

Niben101Scf11738g00026.1 or NbD047748.1

WT: TCTCCCAGGTCACCCAAGT-CCCTGATATCAACATATTCA
*tnp-q1*: TCTCCCAGGTCACCCAAGTTCCCTGATATCAACATATTCA
frameshift leading to stop after aa 51
*tnp-q2*: TCTCCCAGGTCACCCAAGT-- (71 bp deletion) --
frameshift leading to stop after aa 66

Niben101Scf04988g02021.1 or NbD031432.1

WT: CCAGGTCACCCAAG--------------------TCCCTGATATCAACATAT
*tnp-q1*: CCAGGTCACCCAAG-(231 bp insertion)-TCCCTGATATCAACATAT
stop after aa 79
*tnp-q2*: CCAGGTCACCCAAGC-------------------TCCCTGATATCAACATAT
frameshift leading to stop after aa 51

**Supplemental Figure S12. Mutant alleles of *Marchantia polymorpha* and *Nicotiana benthamiana tnp* lines.**
**(A)** Representation of CRISPR/Cas9 mutant *tnp* lines in *M. polymorpha*. Two sgRNA sites targeting the single *TNP* gene in *M. polymorpha* are indicated with arrows. Induced mutations are shown as alignments to the WT sequence. The two independent lines represent independent mutants. **(B)** CRISPR/Cas9 *tnp* mutant lines in *N. benthamiana*. One sgRNA site targeting each of the four *TNP* genes in *N. benthamiana* is indicated with arrows. Induced mutations are shown as alignments to the WT sequence. The two independent lines are homozygous quadruple mutants. Predicted effects of mutations on the protein sequences are shown for both panels.
